# Piezo1‐mediated unfolded protein response in goblet cells regulates intestinal mucus barrier homeostasis through the Ca^2^^+^–IRE1α–XBP1 pathway

**DOI:** 10.1002/ctm2.70886

**Published:** 2026-10-27

**Authors:** Jiandi Wu, Shaotong Wang, Zhe Wang, Jianhua Fang, Xueyan Li, Qingqing Zhang, Yan Dai, Wenjing Tian, Yize Ma, Gangping Li, Lei Zhang, Yudong Jiang, Xiaohua Hou, Jun Song

**Affiliations:** ^1^ Department of Gastroenterology Union Hospital Tongji Medical College Huazhong University of Science and Technology Wuhan China

**Keywords:** mucus barrier, dextran sulphate sodium, water avoidance stress, Piezo1, unfolded protein response, IRE1α, XBP1

## Abstract

**Background:**

Our previous study revealed that Piezo1 in colonic goblet cells senses mechanical stimuli to regulate the production and secretion of MUC2, but the underlying mechanism remains unclear. Altered endoplasmic reticulum function and the unfolded protein response (UPR), which are critical for MUC2 protein folding and secretion, were observed in goblet cell‐specific Piezo1 knockout mice. This study aimed to investigate whether and how Piezo1 in goblet cells modulates mucus barrier function by regulating the UPR.

**Methods:**

In vitro, LS174T cells were subjected to orbital shaking to simulate fluid shear stress (FSS) stimulation. The expression of UPR‐related molecules and MUC2 was detected using real‐time PCR, Western blotting, and immunofluorescence staining in the presence or absence of Piezo1 and UPR inhibition. In vivo, goblet cell‐specific Piezo1 knockout (cKO) mice were treated with dextran sulphate sodium (DSS) and water avoidance stress (WAS). We analysed changes in goblet cell morphology, mucus layer thickness, UPR‐related protein expression and ER structure. In addition, DSS‐induced colitis mice were intraperitoneally injected with Yoda1 to activate Piezo1, and alterations in pathological structures and mucus layer thickness were subsequently evaluated.

**Results:**

Compared with PERK or ATF6, FSS activated PIEZO1 and promoted calcium influx, thereby maintaining ER calcium homeostasis and selectively activating the IRE1α–XBP1 signalling pathway. This cascade facilitated MUC2 production, whereas pharmacological inhibition of PIEZO1 or IRE1α expression significantly abolished these effects. Compared with WT mice, cKO mice exhibited aggravated mucus barrier impairment, downregulated IRE1α‐XBP1 and aggravated ER swelling under DSS and WAS. In contrast, pharmacological activation of Piezo1 via Yoda1 treatment restored intestinal mucus layer thickness and ameliorated colitis in DSS mice.

**Conclusions:**

Piezo1 senses mechanical stimulation to maintain ER calcium homeostasis and modulates the UPR via the IRE1α–XBP1 pathway, thereby facilitating MUC2 production in goblet cells and maintaining intestinal mucosal barrier homeostasis.

**Key points:**

- Piezo1 in colonic goblet cells senses intestinal mechanical stimulation and modulates endoplasmic reticulum calcium homeostasis via Ca^2+^ influx.
- The Piezo1‐Ca^2+^‐IRE1α‐XBP1 signaling axis controls Mucin2 synthesis and production to maintain intestinal mucus barrier integrity.
- Pharmacological activation of Piezo1 by Yoda1 rescues mucus barrier defects, providing a potential therapeutic strategy for gastrointestinal mucosal injury.

## Graphical abstract

This study demonstrates that activation of Piezo1 channels in goblet cells by intestinal mechanical stimulation modulates ER calcium homeostasis via calcium ion (Ca^2^^+^) flux, which in turn regulates Mucin2 production and synthesis through the IRE1α‐XBP1. Intraperitoneal administration of Yoda1 alleviates mucus barrier impairment in DSS/WAS‐treated mice. This axis mechanistically links mechanical sensation to ER function during intestinal mucus barrier biogenesis, uncovering a novel therapeutic target for mucus repair across multiple gastrointestinal disorders.

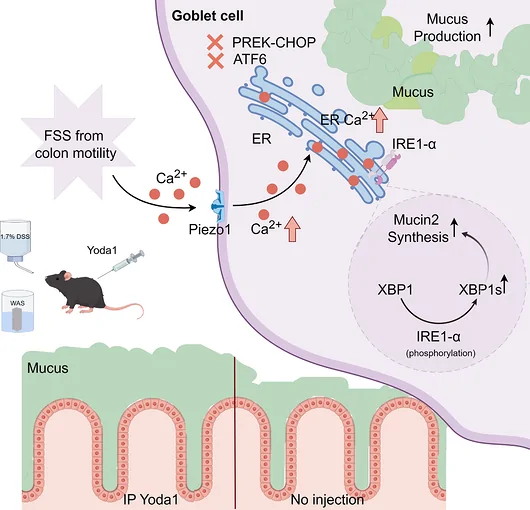

## 1 INTRODUCTION

The intestinal mucus layer, a pivotal protective barrier lining the gut epithelium, is predominantly composed of water, mucins, lipids, proteins and electrolytes. Among these components, mucin‐2 (MUC2), which is secreted by goblet cells (GCs) serves as a core structural element.^1^, ^2^, ^3^, ^4^ GCs dynamically regulate mucus secretion in response to a spectrum of physical, chemical, and biological stimuli.^5^, ^6^, ^7^, ^8^ Impairments in GC function mucus layer integrity are closely linked to a range of digestive disorders, including inflammatory bowel disease (IBD) and irritable bowel syndrome (IBS).^9^, ^10^ However, given that intestinal mucus barrier dysfunction in both conditions is closely linked to goblet cell activity, a critical unanswered question arises: what regulatory mechanisms drive goblet cell‐mediated mucus barrier dysfunction in the two disease states?

In goblet cells, the endoplasmic reticulum (ER) is the pivotal organelle responsible for the synthesis, folding and quality control of mucins. ER homeostasis is tightly linked to mucin processing efficiency and functionality.^11^ Given the extremely high biosynthetic rate of mucins, their production inherently disturbs the ER folding balance and induces ER stress, which triggers the UPR. The UPR maintains mucin homeostasis via key protective mechanisms: upregulating ER‐resident chaperones to increase the folding capacity and mediating ER‐associated degradation (ERAD) to clear misfolded mucins. These actions ensure the efficient maturation and secretion of functional mucins.^12^ Conversely, defective UPR signalling disrupts ER homeostasis, causing misfolded mucins to accumulate and thereby impairing mucin secretion. Such secretion defects ultimately compromise the barrier function of the intestinal mucus layer.^12^

A direct illustration of the functional linkage between the UPR and mucus barrier maintenance is provided by genetic studies. For example, targeted genetic ablation of X‐box binding protein 1 (XBP1), a master transcription factor and key mediator of the UPR, in intestinal epithelial cells (IECs) results in two pivotal phenotypes: a significant downregulation of MUC2 expression and a marked disruption of mucus barrier integrity. These findings provide compelling in vivo evidence that intact UPR signalling via XBP1 is indispensable for sustaining mucus barrier function.^13^ Despite these advances, a critical gap remains in our understanding of intestinal barrier homeostasis: the specific upstream signalling molecules that trigger the activation of the UPR pathway in goblet cells under both physiological and pathological conditions and subsequently modulate mucin secretion have not been fully elucidated.

Piezo1, a mechanically gated nonselective cation channel, mediates calcium ion (Ca^2^^+^) influx into the cytoplasm in response to mechanical stimuli.^14^ While our previous studies revealed that it can regulate MUC2 secretion, the specific underlying mechanism remains unclear.^5^ Emerging evidence reveals cell type‐specific roles of Piezo1 in the intestine. In intestinal epithelial cells, Piezo1‐dependent Ca^2^^+^ influx induces mitochondrial dysfunction and NLRP3 inflammasome activation to promote inflammation,^15^ whereas conditional Piezo1 knockout in enterochromaffin (EC) cells reduces serotonin (5‐HT) secretion, impairing intestinal peristalsis and gastrointestinal transit.^16^ These findings confirm that Piezo1 is critical for regulating cellular physiology via Ca^2^^+^ signalling. However, the ER is the primary intracellular Ca^2^^+^ store, and whether it is modulated by Piezo1 via Ca^2^^+^ signalling to affect mucin secretion in goblet cells remains unclear.

Here, we focus on alterations in Piezo1 expression in goblet cells upon mechanical stimulation, aiming to clarify (1) whether Piezo1 affects the function of the mucus barrier through the UPR, as well as the specific underlying mechanisms, and (2) the role of this Piezo1–ER–mucin regulatory cascade in IBD and IBS models.

## 2 MATERIALS AND METHODS

### 2.1 Mouse models

All experimental mice used in this study were male C57BL/6 mice, purchased from Shanghai Model Organisms Center, Inc. (SMOC). Tamoxifen‐inducible intestinal goblet cell‐specific Piezo1 conditional knockout (cKO) mice (Muc2‐CreERT2; Piezo1^flox/flox^) were generated using a previously described protocol^10^ (goblet cell–specific Piezo1 conditional knockout mice project completion report). Ten 6‐ to 8‐week‐old male wild‐type mice and five cKO mice were randomly selected through random number table for the experimental groups. Among these mice, five WT mice and all five cKO mice were administered 1.7% dextran sulphate sodium salt (MP Biologicals) in the drinking water for 5 consecutive days, followed by a 2‐day observation period without DSS exposure. During the DSS‐induced colitis modelling period, the following parameters were recorded daily: body weight change (calculated as the percentage of the initial body weight), faecal characteristics (scored as normal, loose, or watery), and the presence of haematochezia (scored as absent, occult, or gross). The disease activity index (DAI) was computed using a validated scoring system,^17^ with scores ranging from 0 to 12 (combining body weight loss, faecal consistency and haematochezia). Yoda1 intervention protocol: During 5 consecutive days of DSS administration, mice in the Yoda1‐DSS group received daily intraperitoneal injection of Yoda1 (50 µmol/L; Solarbio) at a dosage of 4 mL kg^−^^1^ day^−^^1^ (100 µL per 25 g body weight). Littermate DSS control mice were injected with an equal volume of normal saline. No mortality was observed among the mice treated with these interventions, with the entire murine cohort being enrolled in the current research. Investigators performing the data analysis were unaware of the allocation status of the experimental mice.

The IBS model was established using a previously described protocol.^10^ Mice from different groups were individually subjected to chronic water avoidance stress (WAS) stimulation. Briefly, each mouse was placed on a 10 cm‐high platform inside a custom‐made acrylic box (30 × 30 × 50 cm^3^) at an ambient temperature of 25°C. The area beneath the platform was filled with water to create an aversive environment. This WAS treatment was performed for 2 h per day (09:00–11:00) for a total of 10 consecutive days.

### 2.2 Evaluation of animal models

#### 2.2.1 Visceral hypersensitivity assessment

Prior to sacrifice, mice underwent abdominal withdrawal reflex (AWR) scoring. A sterile catheter was inserted 2.0 cm into the anus and fixed to the tail base. After 10 min of acclimation, graded air pressure (20, 40, 60 and 80 mmHg) was delivered via a microinjection pump (20 s/stimulation, 5‐min intervals). Behavioural responses were scored (0–4) by two observers who were blinded to the groups, with each pressure tested in triplicate to calculate the mean values.

#### 2.2.2 Tissue collection and histology

Mice were euthanized by cervical dislocation. The entire colon was isolated and cleaned of adherent tissue, and its length was measured with digital callipers. A 0.5 cm segment from the colon (≈2 cm from the cecum and anus) was fixed with 4% paraformaldehyde (4°C, 24 h), embedded in paraffin, sectioned (5 µm), and stained with hematoxylin and eosin (HE). Histopathological scoring (0–12 total) was performed by two pathologists who were blinded to the groups and who evaluated mucosal damage, inflammatory cell infiltration and gland destruction (0–4 points each).

#### 2.2.3 Analysis of the mucus layer

Another 0.5 cm distal colon segment was fixed with Carnoy's solution (room temperature, 4 h), embedded in paraffin, sectioned, and stained with Alcian blue (Solarbio, G1563). The mucus layer thickness was imaged under a light microscope (100× or 200× magnification) and quantified using ImageJ software, with 2–3 typical nonoverlapping fields averaged per section.

### 2.3 Single‐cell RNA‐seq and analysis

Colons were harvested from WT and cKO C57BL/6 mice, with 3 animals per genotype. The entire colon, spanning from the caecocolic junction to the anus, was processed to generate a single‐cell suspension.

The designated cells were sorted into PBS according to the DNBelab C‐TaiM4 protocol. The resulting cell suspension was loaded into a DNBelab C4 (BGI), and single‐cell cDNA libraries were then constructed using a DNBelab C Series High‐throughput Single‐Cell Library Preparation Kit (MGI, 940‐000047‐00) according to the manufacturer's protocols. The libraries were quantified and subjected to 150 bp paired‐end sequencing on a DNBSEQ‐T7 instrument (BGI). Raw base call files were demultiplexed using the 10x Genomics Cell Ranger pipeline and aligned to the GRCm39 transcriptome. Single‐cell RNA‐seq data were processed for downstream analysis in Python (v3.10.6) using the Scanpy package, with the following steps: quality control and filtering of retained cells expressing > 300 genes, exclusion of those with mitochondrial gene counts > 25% (to remove low‐quality cells/debris), and elimination of doublets using the Scrublet algorithm. Normalization included log‐transformation of gene expression counts per cell; the top 2000 highly variable genes were selected for subsequent dimensionality reduction and clustering. Principal component analysis (PCA) was performed on scaled highly variable gene data, followed by batch effect correction using Harmony. Corrected embeddings were used for nonlinear dimensionality reduction (UMAP) and clustering. Cells were clustered with the Leiden algorithm: initial low‐resolution clustering was followed by isolation of Epcam+ epithelial cells, which were reclustered at higher resolution. Clusters were manually annotated to cell types on the basis of canonical marker gene expression. Differentially expressed genes (DEGs) were identified using the Wilcoxon rank‐sum test (*p* < .05, |log2FC| > .5) and visualized in volcano plots. DEGs were subjected to enrichment analyses using the Gene Ontology (GO) and Kyoto Encyclopedia of Genes and Genomes (KEGG) databases to interpret biological functions/pathways. The figures were generated using https://www.bioinformatics.com.cn.

### 2.4 RNA‐Seq and bioinformatics analyses of LS174 T cells

Total RNA was extracted from LS174T cells according to the manufacturer's protocol. cDNA was synthesized via reverse transcription and then amplified by PCR after repair. Amplicons were purified using Ampure XP beads, eluted in EB buffer and quantified. The purified double‐stranded PCR products were heat denatured and circularized to construct the final library. DNA nanoballs (DNBs) were prepared, and sequencing was performed on the DNBSEQ‐T7 platform (BGI Genomics). KEGG and GO analyses were performed as previously described. The RNA‐Seq data used in this study are available from the corresponding author upon reasonable request. These data cannot be deposited in a public repository, as they include components associated with ongoing unpublished work from other related studies.

### 2.5 Electron microscopy sample preparation and observation using

#### 2.5.1 Scanning electron microscopy (SEM)

The distal segment of the colon tissue was fixed with a 2.5% glutaraldehyde solution at 4°C for 24 h. After gradient dehydration with ethanol (30%, 50%, 70%, 90%, 95% and 100%), the samples were dried using a critical point dryer (Quorum Technologies, K850). The dried samples were subsequently sputter‐coated with gold/palladium and observed under a scanning electron microscope (HITACHI, SU8100). Images were captured and analysed for morphological characterization.

#### 2.5.2 Transmission electron microscopy (TEM)

Colon tissue samples were initially fixed with 2.5% glutaraldehyde (the same as for SEM fixation, for consistency) at 4°C overnight. After three rinses with 0.1 M phosphate‐buffered saline (PBS, pH 7.4), the samples were postfixed with 1% osmium tetroxide (OsO_4_) dissolved in 0.1 M PBS at room temperature for 2 h in the dark. The samples were then dehydrated through a graded ethanol series (30% to 100%) followed by infiltration with propylene oxide. Next, the samples were embedded in epoxy resin (Epon 812) and polymerized at 60°C for 48 h. Ultrathin sections (60–80 nm) were cut using an ultramicrotome, stained with uranyl acetate and lead citrate for contrast enhancement, and finally observed under a transmission electron microscope (HITACHI, HT7800). Representative images were acquired for ultrastructural analysis. ImageJ software was applied to quantify the ER and cytoplasmic areas from the TEM images. Multiple typical ER regions were measured and averaged per group, and the ER/cytoplasmic area ratio was presented as the bubble size in the scatter plot to evaluate ER swelling among the three groups.

#### 2.5.3 Immunofluorescence Staining

Mid‐colon segments were fixed with 4% paraformaldehyde, embedded in paraffin, and sectioned (5–8 µm). After dewaxing in xylene and rehydration through graded ethanol solutions, the sections were blocked with 5% donkey serum (Antgene, 050715) for 30 min at 37°C. The sections were incubated with primary antibodies overnight at 4°C, followed by incubation with fluorescent secondary antibodies (Yeasen Biotechnology, 34906ES, 1:200; Servicebio, GB21403, 1:200) for 1 h at room temperature in the dark. Nuclei were counterstained with DAPI (1:1000 dilution; Antgene, ANT063), and images were captured using a fluorescence microscope (ZEISS Axio Observer5). Immunofluorescence images were analysed using ImageJ software for quantitative assessment. The number of MUC2‐positive cells (green fluorescence) and total cell nuclei labelled by DAPI (blue fluorescence) were counted individually, and the Muc2 goblet‐cell‐positive area was quantified and normalized to the tissue area (positive area/mm^2^ tissue section), with 2–3 typical nonoverlapping fields averaged per section. For co‐immunofluorescence staining, colocalization between green and red signals was assessed qualitatively by merged yellow fluorescence, whereas protein quantification was achieved primarily using Western blotting. The following primary antibodies were used: AGR2 (1:200, R&D Systems, AF6068), p‐IRE1 (1:200, R&D Systems, NB100‐2323), sXBP1 (1:200, Proteintech, 24868‐1‐AP), PIEZO1 (1:200, Proteintech, 15939‐1) and MUC2 (1:200, GeneTex, GTX100664).

#### 2.5.4 Protein sampling and detection

Colonic epithelial tissue or harvested cells were lysed in 100 µL of RIPA buffer supplemented with 1% protease inhibitor cocktail (Servicebio, G2008‐1ML) and phosphatase inhibitors (Servicebio, G2007‐1ML). The samples were ground and incubated on ice for 30 min to ensure complete lysis. After centrifugation at 12 000 × g at 4°C for 15 min, the supernatant was collected as total protein.

#### 2.5.5 Western blot analysis

Protein concentrations were determined using a BCA protein assay kit (Bosetr, AR1189). Equal amounts of protein were mixed with loading buffer (Servicebio, G2075‐10ML) and denatured by boiling for 10 min. Standard Western blot procedures were followed, including sodium dodecyl sulphate–polyacrylamide gel electrophoresis (SDS–PAGE), electrotransfer to polyvinylidene difluoride (PVDF) membranes, antibody incubation and chemiluminescence detection. Electrotransfer was performed under ice‐cold conditions, with the duration adjusted according to the molecular weight of the target protein. For targets with overlapping molecular weights, membranes were stripped using a stripping buffer (Beyotime, P0025), reblocked, and reprobed with antibodies against other proteins. Western blot band intensity was analysed using ImageJ software (National Institutes of Health). The antibodies used were as follows: Piezo1 (1:1000, ABclonal, A4340SP), IRE1α (1:1000, ABclonal, A21021), p‐IRE1α (1:1000, Abcam, ab124945), sXBP1 (1:1000, ABclonal, A222546), sXBP1 (1:1000, Proteintech, 24868‐1‐AP), PERK (1:1000, Proteintech, 24390‐1‐AP), p‐PERK (1:1000, Proteintech, 29546‐1‐AP), ATF6 (1:1000, Abcam, ab24169), β‐tubulin (1:1000, Beyotime, AF1216), GAPDH (1:1000, Beyotime, AF1186), CHOP (1:1000, ABclonal, A20987) and GRP78 (1:1000, GeneTex, 113340).

#### 2.5.6 Enzyme‐linked immunosorbent assay (ELISA)

To measure TNF‐α and IL‐1β levels, total protein extracted from intestinal tissues was diluted 10‐fold, and the expression levels were measured using commercial ELISA kits (TNF‐α: ABclonal, RK04875; IL‐1β: ABclonal, RK04878) according to the manufacturers’ instructions. To measure Muc2 levels in cell supernatants, the treated cell supernatants were centrifuged at 1000 × g for 20 min to remove impurities and cell debris, and Muc2 levels were subsequently measured using an ELISA kit (ELK Biotechnology, ELK2010) according to the manufacturer's protocol.

#### 2.5.7 Real‐time quantitative reverse transcription polymerase chain reaction (RT–qPCR)

RT–qPCR was performed using a LightCycler 480 instrument (Roche) according to previously described methods.^18^ Briefly, reactions were prepared with SYBR Green qPCR Master Mix (ChamQ SYBR qPCR Master Mix; Invitrogen, Q311) according to the manufacturer's protocol. Gene‐specific primer pairs were used to amplify the transcripts of target genes, including IRE1α, sXBP1, MUC2 and PIEZO1, under optimized reaction conditions. Glyceraldehyde‐3‐phosphate dehydrogenase (GAPDH) served as the housekeeping gene for normalization. Relative gene expression levels were calculated using the 2^(‐ΔΔCt)^ method. The primers used were as follows: hPIEZO1 CCGCTCGTTTCCGAGTCAC and TGGTAGCAGTAGAGGCAGATG; hEDEM1 CGGACGAGTACGAGAAGCG and CGTAGCCAAAGACGAACATGC; hGAPDH TGCACCACCAACTGCTTAGC and GGCATGGACTGTGGTCATGAG; hMUC2 CAGCACCGATTGCTGAGTTG and GCTGGTCATCTCAATGGCAG. h‐IRE1a CACAGTGACGCTTCCTGAAAC and GCCATCATTAGGATCTGGGAGA; h‐sXBP1 AACCTGTAGAAGATGACCTCGTTCC and AAAGAGTTCATTGGCAAAAGTGTCC; mMuc2 TCCACATTATCACCTTGAC and AGGGAATCGGTAGACATC; and mGAPDH AGGTCGGTGTGAACGGATTTG and TGTAGACCATGTAGTTGAGGTCA.

### 2.6 Cell culture and interventions

LS174T cells were cultured, and Piezo1 knockdown was performed as previously described.^5^ Control cells were subjected to identical lentiviral manipulations using an empty vector. Cells were seeded in 6‐well or 12‐well plates, and mechanical stimulation was applied using established protocols.^5^ For FSS stimulation, culture plates were placed on a shaker to generate bidirectional vertical oscillation, with stimulation delivered at 30 cycles/min for 24 h. This shaking regimen was designed to simulate intestinal luminal fluid shear stress stimulation, a physiological mechanical force sensed by intestinal epithelial cells in vivo.

For inhibitor treatments:

- The cells were pretreated with BAPTA‐AM (10 µmol/L; MCE, HY‐100545) for 30 min prior to mechanical stimulation (or as a no‐treatment control).
- GsMTx4 (0.1 µmol/L; MCE, HY‐P1410) and 4μ8C (100 µmol/L; MCE, HY‐19707) were added concurrently with mechanical stimulation.

For activator treatments:

- The cells were treated with Yoda1 (75 µmol/L; Solarbio, IY0290) for 24 h.
- The cells were treated with Tunicamycin (2 µg/mL; Solarbio, IT2670) for 24 h.
- The cells were treated with Cinnamaldehyde (30 µmol/L; Solarbio, IC1140) for 24 h.

#### 2.6.1 siRNA‐mediated IRE1α knockdown

Cells were seeded in 1 mL of antibiotic‐containing medium and transfected upon reaching 70% confluence. Pepincell™ Transfection Reagent (GENE CREATE) was added to the serum‐free medium and incubated for 5 min. Separately, the siRNA targeting IRE1α was diluted in serum‐free medium and incubated for 5 min. The two mixtures were combined, incubated at room temperature for 15–20 min, and added to cells maintained in serum‐free medium. After incubation for 4–6 h at 37°C, the medium was replaced with complete medium. mRNA expression was assessed at 24–72 h post‐transfection, and protein expression was analysed at 48–96 h post‐transfection. The sequences of the siRNAs targeting human IRE1α (hIRE1α‐1769) were as follows: sense, 5′‐CUGGAACAAGACGAUGGAGAUTT‐3′; and antisense, 5′‐AUCUCCAUCGUCUUGUUCCAGTT‐3′.

#### 2.6.2 Calcium ion detection

The ER calcium indicator plasmid (CMV‐ER‐LAR‐GECO1) was purchased from Miaoling Plasmid (P18477) and transfected into cells using the aforementioned transfection protocol.

For cytoplasmic Ca^2^^+^ detection, approximately 1 × 10^6^ washed cells were collected, resuspended, and incubated with FLUO‐4 AM (Solarbio, IF1500) at a final concentration of 1 µmol/L. After being incubated at 37°C for 1 h, the cells were washed three times with PBS to remove the excess dye. Cytoplasmic and ER Ca^2^^+^ levels were determined by flow cytometry using a BD FACSymphony™ A1 instrument (Becton, Dickinson and Company), and the acquired data were subsequently analysed with FlowJo_V10.8.1 software. Calcium ion immunofluorescence confocal imaging was performed on a Nikon AX R confocal microscope equipped with an NSPARC (Nikon Spatial Array Confocal) detector.

#### 2.6.3 Coimmunoprecipitation (CoIP)

Total protein was extracted as described above. The magnetic beads were thoroughly resuspended, and 25 µL of beads (MedChemExpress, HY‐K0202‐1 mL) were transferred to a 1.5 mL microcentrifuge tube. Beads were washed by adding 400 µL binding/washing buffer, resuspended thoroughly, and separated on a magnetic rack; the supernatant was discarded. For antibody conjugation, 400 µL of diluted anti‐Piezo1 antibody (Alomone Labs, APC‐087) was added to the washed beads, which were then resuspended completely and incubated on a rotary shaker (30 min at room temperature, followed by 2 h at 4°C). After incubation, the beads were collected via magnetic separation and washed with binding/washing buffer. Next, 400 µL of prepared protein lysate (antigen sample) was added to the antibody‐conjugated beads, which were then resuspended thoroughly and incubated again on a rotary shaker (30 min at room temperature, followed by 2 h at 4°C). The beads were then separated magnetically, the supernatant was discarded, and the beads were subsequently washed 4 times with binding/washing buffer. Finally, the beads were resuspended in 25–50 µL of 1× SDS–PAGE loading buffer, mixed thoroughly, and heated at 95°C for 5 min to elute immune complexes. After magnetic separation, the supernatant was collected for SDS–PAGE analysis.

### 2.7 Statistical analysis

Statistical analyses were performed and histograms were plotted using GraphPad v9.0. All the data are presented as the means ± standard errors of the means (SEMs). The Shapiro–Wilk test was applied to assess data normality. For normally distributed data, comparisons between two groups were analysed using the unpaired t test, whereas multigroup comparisons were conducted using one‐way analysis of variance (ANOVA). When significant differences were identified, Tukey's post hoc test was further used for pairwise comparisons. For nonnormally distributed data, the Mann–Whitney U test (for two groups) or the Kruskal–Wallis H test (for multiple groups) was employed.

## 3 RESULTS

### 3.1 Result 1: The knockout of Piezo1 in goblet cells does not induce pathological intestinal damage but does trigger low‐grade inflammation, goblet cell swelling, and impaired mucus production

We generated cKO mice to investigate the role of Piezo1 in goblet cells. The detailed generation procedures are shown in Figure 1A. Although the body weight of cKO mice tended to decrease during feeding, the difference was not statistically significant (Figure 1B). Immunofluorescence colocalization assays using the goblet cell marker Agr2 and Piezo1 protein confirmed the successful ablation of Piezo1 in goblet cells (Figure 1C). Histopathological analysis of the colon revealed no overt pathological lesions in the cKO mice (Figure 1D), and immunofluorescence staining revealed no significant changes in the expression of the tight junction protein ZO‐1, a key marker of intestinal epithelial barrier function (Figure S1). Nevertheless, the protein levels of the proinflammatory cytokines IL‐1β and TNF‐α increased in intestinal tissues, which indicated low‐grade inflammation (Figure 1E,F). Further analyses revealed a reduced thickness of the intestinal mucus layer (Figure 1G,K) and a decreased number of goblet cells in the colons of cKO mice (Figure 1H,L). Transmission electron microscopy revealed that Piezo1 ablation induced prominent swelling of the goblet cells, which was accompanied by a tendency to detach from the intestinal villi (Figure 1I,J). Additionally, the RT–qPCR results verified the significant downregulation of Muc2 gene expression (Figure 1M). After treating the goblet cell lines with Yoda1, we subsequently analyzed the correlation between PIEZO1 and MUC2 expression. The results revealed a positive correlation between these two genes (Figure 1N). However, the specific molecular mechanisms mediating these phenotypic changes remain to be further elucidated.

**FIGURE 1:**
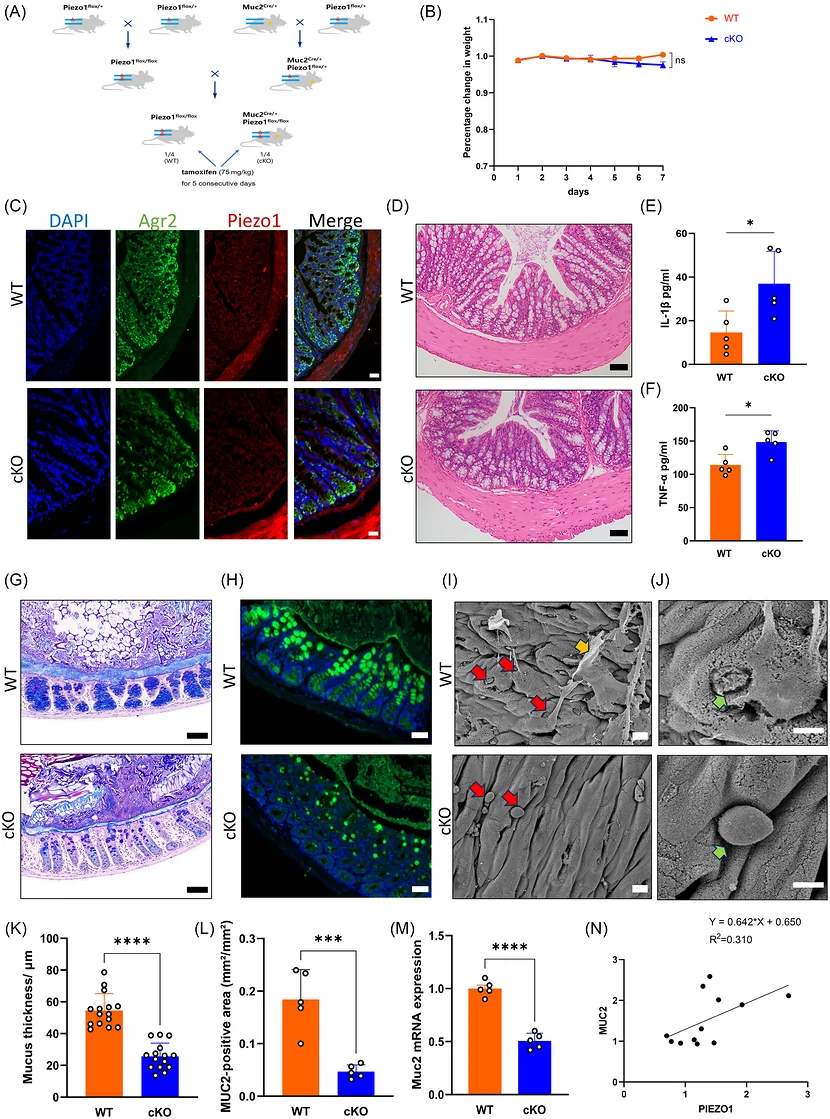
Pathological changes and mucus layer phenotypes in goblet cell‐specific Piezo1 knockout mice. (A) Schematic illustration of mouse breeding scheme for generating cKO mice and littermate WT controls, with tamoxifen induction protocol. (B) Body weight percentage change of WT and cKO mice within 7 days after tamoxifen treatment. (C) Immunofluorescence costaining of the colonic goblet cell markers Agr2 and Piezo1 in WT and cKO mice. Scale bar: 20 µm, 400× magnification. (D) Hematoxylin–eosin (HE) staining of pathological colonic sections. Scale bar: 50 µm, 200× magnification. (E and F) ELISA results showing the levels of IL‐1β (E) and TNF‐α (F) in colon tissues. (G and K) Alcian blue–periodic acid–Schiff (AB–PAS) staining of colonic tissues (G) and corresponding quantitative analysis (K). Two to three representative images of the mucus layer were captured from each mouse for measurement (*n* = 5). Scale bar: 100 µm, 100× magnification. (H and L) Immunofluorescence staining for Muc2 in colon tissues (H) and the corresponding quantitative analysis (L); *n* = 5. Scale bar: 50 µm, 200× magnification (for F). (I and J) Scanning electron microscopy (SEM) images of colonic goblet cells (I, 1000× magnification; Scale bar: 10 µm) and a locally magnified view (J, scale bar: 5 µm); goblet cells are indicated by red arrows and green arrows, while intestinal mucus is marked by yellow arrows. (M) qRT–PCR analysis of Muc2 mRNA expression in intestinal epithelial tissues from WT and cKO mice (*n* = 5). (N) Linear regression analysis of the correlation between PIEZO1 and MUC2 mRNA levels in LS174T cells treated with or without Yoda1. X‐ and Y‐axes represent relative mRNA expression levels. *p < .05, **p < .01, ***p < .001, and ****p < .0001.

### 3.2 Result 2: Piezo1 knockout impairs endoplasmic reticulum function in goblet cells

We performed single‐cell RNA sequencing (scRNA‐seq) on colonic tissues from WT mice and cKO mice to further explore the regulatory mechanism of Piezo1 in goblet cell function. IECs were isolated and subjected to dimensionality reduction analysis, and uniform manifold approximation and projection (UMAP) plots were generated to visualize cell clusters. A total of 9 distinct intestinal epithelial cell subpopulations were annotated (Figure 2A,B), encompassing 4334 IECs in the WT group and 7263 IECs in the cKO group. We subsequently focused on intercrypt goblet cells to explore the molecular mechanisms through which Piezo1 modulates mucus production from goblet cells. The volcano plot shows that Piezo1 ablation significantly downregulated 790 genes and upregulated 527 genes in these cells (Figure 2C). Kyoto Encyclopedia of Genes and Genomes (KEGG) pathway enrichment analysis highlighted prominent perturbations in ER‐related protein synthesis pathways (Figure 2D). GO enrichment analysis revealed that in the cellular component (CC) category, the expression of rough endoplasmic reticulum, a critical organelle for protein folding and modification, significantly changed; moreover, the biological process (BP) category was characterized by altered regulation of protein modification (Figure 2E). Additionally, the expression of Xbp1 and activating transcription factor 4 (Atf4), key mediators of the UPR pathway, was significantly decreased in goblet cells from cKO mice (Figure 2F).

**FIGURE 2:**
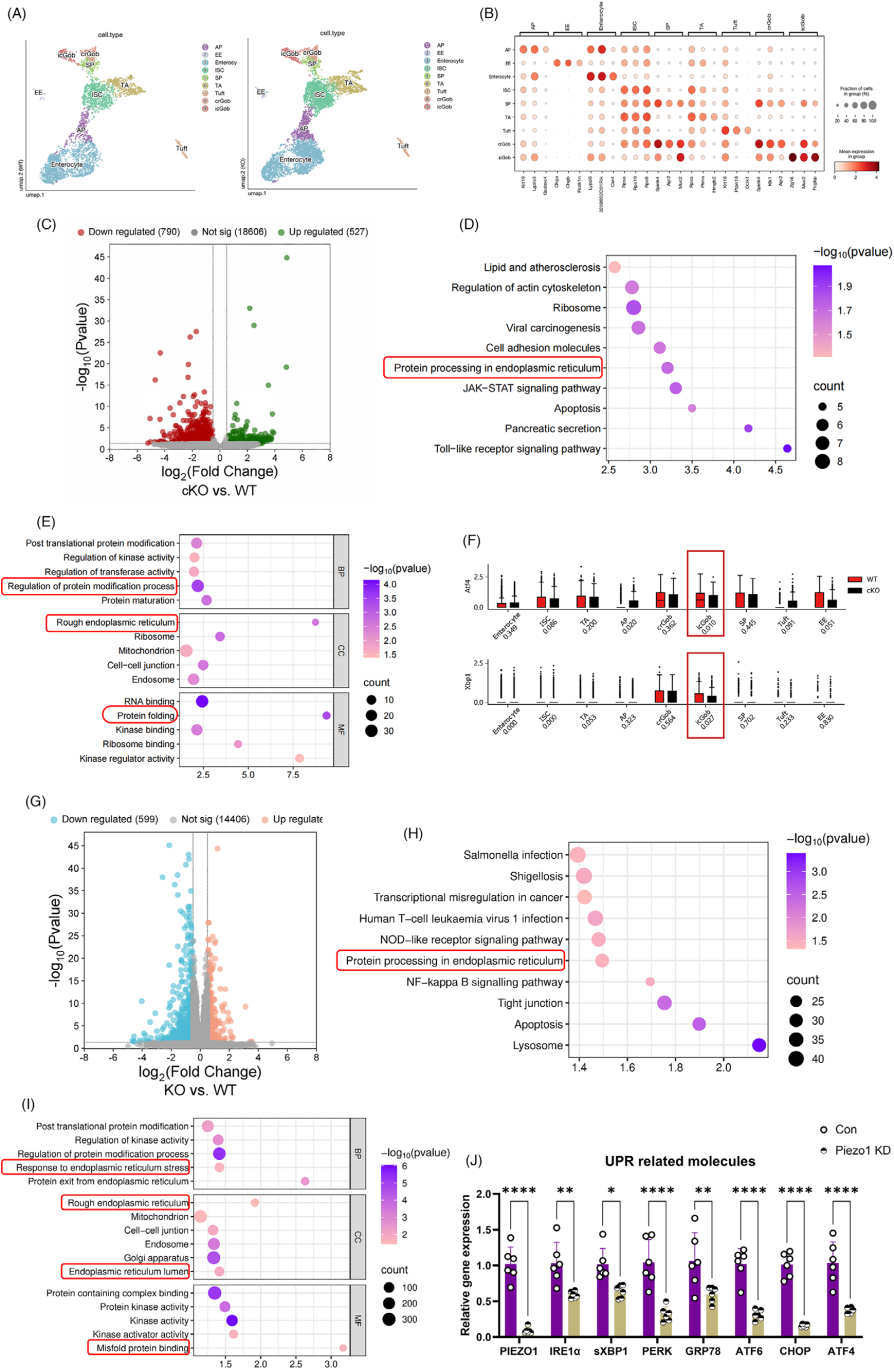
Single‐cell sequencing results for the colon from goblet cell‐specific Piezo1 knockout mice and transcriptome sequencing results for the PIEZO1‐knockdown LS174T cell line. (A) Uniform manifold approximation and projection (UMAP) plot of single‐cell RNA sequencing (scRNA‐seq) data from intestinal epithelial cells isolated from cKO mice (KO) and their WT littermates (3 mice per group). (B) Plot showing marker gene annotations in colonic epithelial cells. (C) Volcano plot showing differential gene expression profiles in intercrypt goblet cells between cKO and WT mice (*n* = 3). (D) Plot of the Kyoto Encyclopedia of Genes and Genomes pathway enrichment analysis illustrating altered signaling pathways in goblet cells following Piezo1 knockout. (E) Plot of the Gene Ontology enrichment analysis (including biological process, cellular component, and molecular function categories) of goblet cells following Piezo1 knockdown. (F) Changes in the expression of Xbp1 and Atf4 in various colonic epithelial cell subsets from WT and cKO mice, ISC, Intestinal Stem Cell; TA, Transit‐Amplifying Cell; AP, Absorptive Progenitor; crGob, Crypt Goblet Cell; icGob, intercrypt Goblet Cell; SP, Secretory Progenitor; Tuft, Tuft Cell; EE, Enteroendocrine Cell. (G) Volcano plot depicting differentially expressed genes in LS174T cells after PIEZO1 knockdown. (H) Plot of the KEGG pathway enrichment analysis showing altered pathways in PIEZO1‐knockdown LS174T cells. (I) Plot of the Gene Ontology enrichment analysis (including biological process, cellular component, and molecular function categories) of PIEZO1‐knockdown LS174T cells. (J) Expression levels of UPR‐related mRNAs in LS174T cells with or without PIEZO1 knockdown. Con, Control; Piezo1 KD, Piezo1 knockdown. * *p* < .05, **p < .01, ***p < .001, and ****p < .0001.

LS174T is a human colorectal cancer cell line derived from intestinal goblet cells. We performed bulk transcriptome sequencing of a Piezo1‐knockout LS174T cell line to validate these findings and mitigate individual variability among mice. A volcano plot revealed 599 downregulated genes and 365 upregulated genes (Figure 2G). Consistent with the scRNA‐seq results, the results of the KEGG and GO analyses confirmed significant alterations in ER protein synthesis and ER stress response pathways (Figure 2H,I). RT–qPCR further verified that IRE1α, sXBP1, PERK, GRP78, ATF6, CHOP and ATF4 expression was significantly downregulated following Piezo1 deletion (Figure 2J). Collectively, these data indicate that Piezo1 knockout in goblet cells induces ER dysfunction. Given that the folding of MUC2 dimers requires a stable ER environment, we propose that PIEZO1 mediates ER function to regulate MUC2 protein synthesis and production, thereby modulating the integrity of the intestinal mucosal barrier.

### 3.3 Result 3: PIEZO1 activation enhances ER function through the IRE1α–XBP1 pathway

We further explored the regulatory mechanism of PIEZO1 in ER‐related molecular functions by applying mechanical stimulation to LS174T cells using a shaker board using a previously described protocol.^10^ FSS stimulation was applied to mechanically stimulate LS174T cells to mimic intestinal shear stress, while Yoda1 was used as a pharmacological agonist to activate Piezo1 channels. Combined treatment with these two interventions was performed to explore their effects on MUC2 production and the expression of UPR‐related molecules. FSS stimulation significantly upregulated PIEZO1 expression (Figure 3A), and RT–qPCR and enzyme‐linked immunosorbent assay results demonstrated that both MUC2 mRNA and protein levels markedly increased, with changes comparable to those associated with Yoda1 intervention (Figure 3B,C). RT–qPCR analysis of ER‐related signalling molecules revealed significant activation of the IRE1α–XBP1 pathway (Figure 3D,E), accompanied by markedly increased expression of its downstream target EDEM1 (Figure 3F). In addition, the activity of the PERK‐CHOP and ATF6 pathways tended to increase (Figure S2a–e). Western blot analysis further validated the robust activation of the IRE1α–XBP1 pathway (Figure 3G–I), whereas no significant alterations were observed in the GRP78, PERK–CHOP, or ATF6 pathways (Figure 3J–N). In addition, immunofluorescence staining revealed that the fluorescence intensities of phosphorylated IRE1α (p‐IRE1α) and sXBP1 significantly increased following FSS stimulation (Figure 3O,P). Western blot detection of key UPR proteins, including GRP78, sXBP1 and CHOP, is frequently hampered by nonspecific bands. To validate the specificity of the target bands in our present study, we performed supplementary experiments with tunicamycin (2 µg/mL, 24 h), a well‐established ER stress inducer. Specific bands of GRP78, sXBP1 and CHOP were markedly induced after tunicamycin stimulation, verifying the credibility of our western blot quantification results (Figure S3a–d). Collectively, these data further suggest that PIEZO1 is activated to regulate MUC2 production specifically through the IRE1α–XBP1 pathway rather than the PERK–CHOP or ATF6 pathway.

**FIGURE 3:**
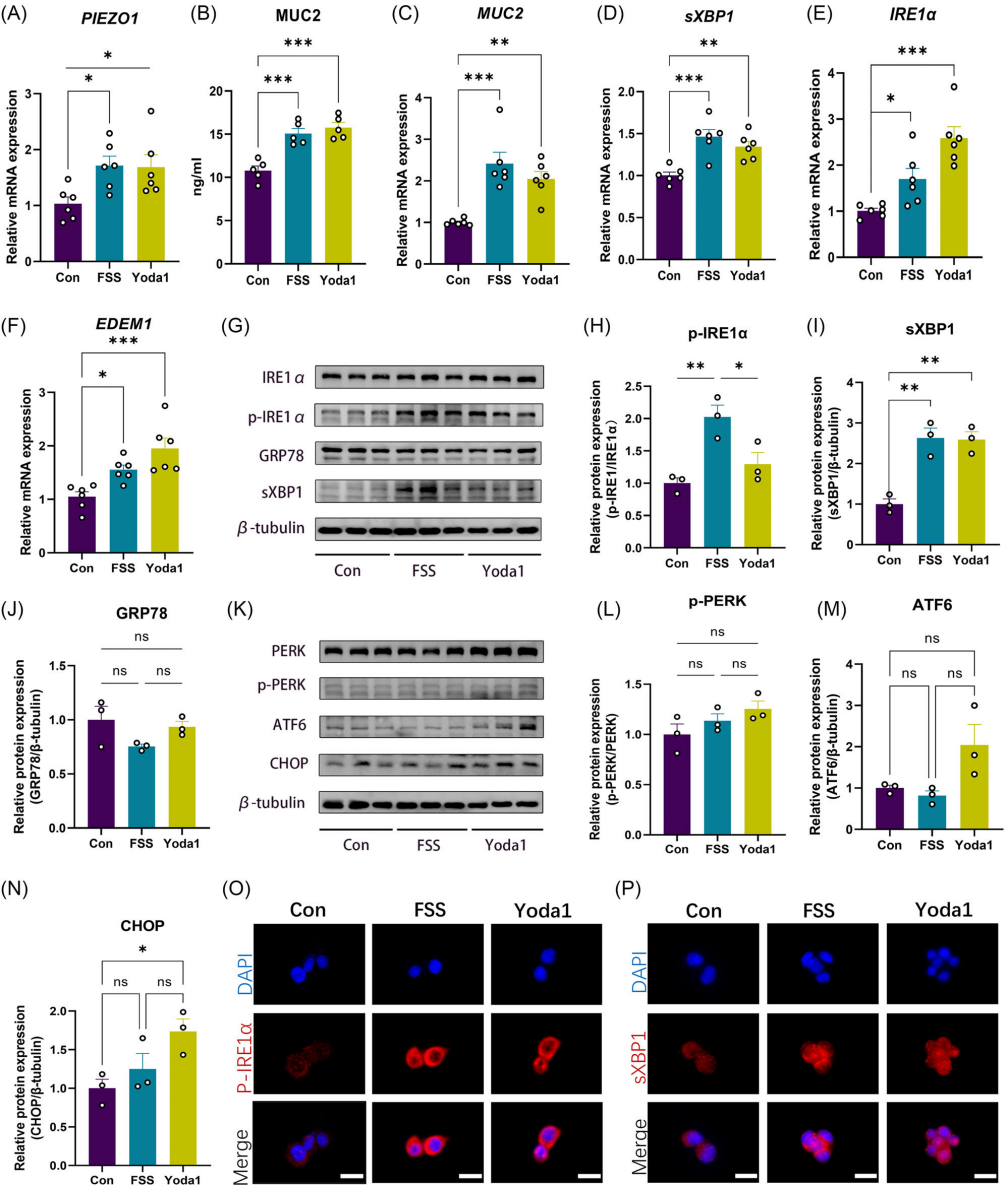
Effects of mechanical stimulation and Yoda1 intervention on PIEZO1‐regulated expression of MUC2 and UPR‐related molecules. (A) Relative mRNA expression level of PIEZO1 measured by qRT–PCR. (B) MUC2 protein level detected using an ELISA. (C–F) qRT–PCR analysis of MUC2 (C), sXBP1 (D), IRE1α (E) and EDEM1 (F) mRNA expression. (G–J) Representative Western blot images and corresponding quantitative analyses of the levels of phosphorylated IRE1α (p‐IRE1α/IRE1α) (G and H), sXBP1 (G and I), and GRP78 (G and J). (K–N) Representative Western blot images and corresponding quantitative analyses of the levels of phosphorylated PERK (p‐PERK/PERK) (K and L), ATF6 (K and M) and CHOP (K and N). (O and P) Immunofluorescence staining for p‐IRE1α and XBP1s in cells (magnification: 400×; scale bar: 20 µm). fluid shear stress (FSS) stimulation, vertical shaking at 30 rpm for 24 h with a shaker to mimic intestinal shear stress. Yoda1, a PIEZO1 agonist, was used at a final concentration of 75 µmol/L for 24 h under static culture prior to cell collection. Means ± SEMs, *n* = 3–6. Con, Control. ns, not significant, *p < .05, **p < .01, ***p < .001, and ****p < .0001.

### 3.4 Result 4: The inhibition or knockout of PIEZO1 abrogates the FSS stimulation induced activation of the IRE1α–XBP1 pathway and attenuates MUC2 production

We subsequently treated mechanically stimulated cells with GsMTx4, a widely used inhibitor of PIEZO1, to confirm that FSS stimulation modulates the IRE1α–XBP1 pathway and MUC2 primarily via PIEZO1 (Figure 4A). GsMTx4 suppressed the FSS stimulation induced upregulation of MUC2 at both the mRNA and protein levels (Figure 4B,F) and reduced the expression of sXBP1 and its downstream target EDEM1 (Figure 4C,D), whereas IRE1α mRNA levels remained unaltered (Figure 4E). However, both immunofluorescence staining and Western blot analyses demonstrated that GsMTx4 inhibited FSS stimulation‐induced IRE1α phosphorylation and sXBP1 protein expression (Figure 4G–K), indicating that IRE1α may exert its downstream effects through its phosphorylated form. We further validated these findings by knocking down PIEZO1 in LS174T cells using shRNA‐mediated lentiviral gene knockdown (Figure 4L). Consistent with the results obtained with the inhibitor, PIEZO1 knockdown attenuated the FSS stimulation‐induced increase in MUC2 expression (Figure 4M) and reversed the upregulation of phosphorylated IRE1α and sXBP1 (Figure 4N–P). These results indicate that FSS stimulation activates PIEZO1, thereby regulating the IRE1α–XBP1 pathway and MUC2 production.

**FIGURE 4:**
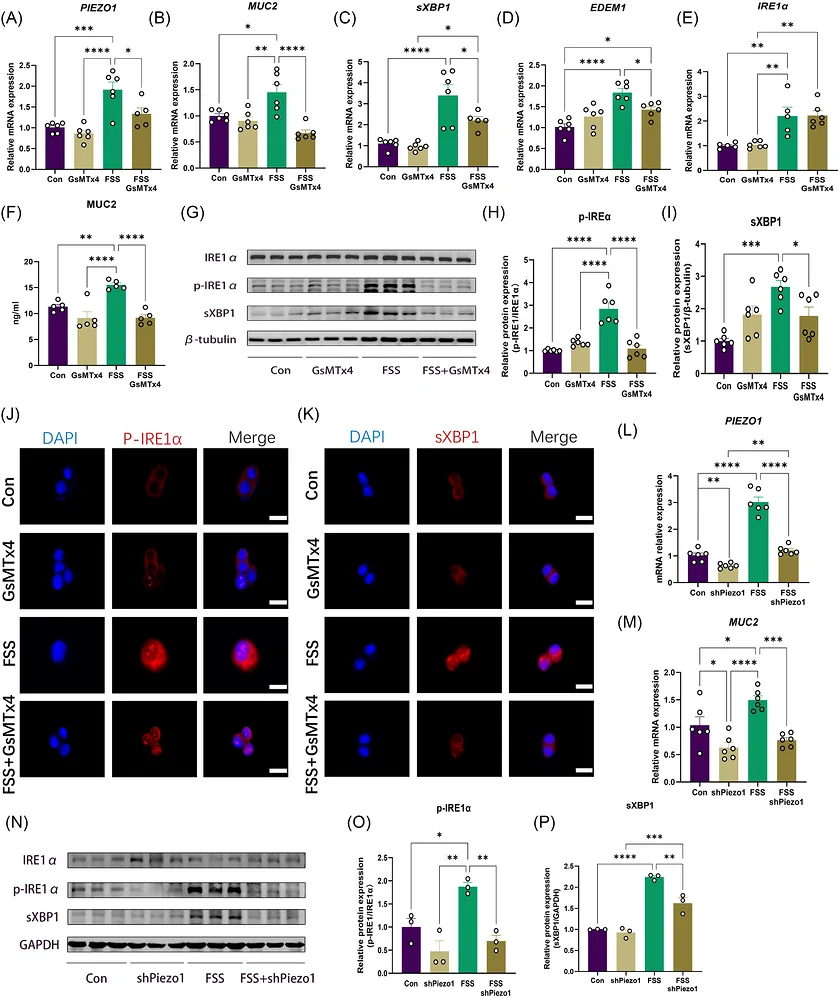
Effects of PIEZO1 inhibition or knockdown on the mechanically induced activation of the IRE1α–XBP1 pathway and MUC2 production. (A–E) qRT–PCR analysis of PIEZO1 (A), MUC2 (B), sXBP1 (C), EDEM1 (D), and IRE1α (E) mRNA expression levels under FSS or no FSS conditions, with or without GsMTx4 treatment (0.1 µmol/L, 24 h). (F) ELISA results showing the MUC2 protein level under the aforementioned experimental conditions. (G–I) Representative western blot images and corresponding quantitative analyses of phosphorylated IRE1α (p‐IRE1α/IRE1α) (G and H) and sXBP1 (G and I) levels in cells treated with or without FSS and GsMTx4. (J and K) Immunofluorescence staining for p‐IRE1α and sXBP1 (magnification: 400×; scale bar: 20 µm) under FSS or no FSS conditions and treatment with or without GsMTx4. (L and M) qRT–PCR analysis of Piezo1 (L) and MUC2 (M) mRNA expression levels under FSS or no FSS conditions, with or without PIEZO1 knockdown. (N–P) Representative Western blot images and corresponding quantitative analyses of p‐IRE1α/IRE1α (N, O) and sXBP1 (N, P) levels in cells treated with or without FSS and shPiezo1‐mediated PIEZO1 knockdown. shPiezo1 indicates lentivirus‐delivered short hairpin RNA that specifically knockdown PIEZO1 expression. Means ± SEMs, *n* = 3–6. ns, not significant, *p < .05, **p < .01, ***p < .001, and ****p < .0001.

### 3.5 Result 5: The inhibition or knockout of IRE1α abrogates the MUC2 production induced by FSS stimulation‐mediated PIEZO1 activation

We next investigated whether targeting IRE1α affects FSS stimulation‐induced MUC2 upregulation, as well as its impact on the downstream proteins sXBP1 and EDEM1. Immunofluorescence staining and Western blot analyses revealed that 24 h of treatment with 4μ8C, a specific inhibitor of IRE1α endoribonuclease activity and kinase activity (Figure 5A,B,H), suppressed the FSS stimulation‐induced increase in MUC2 expression (Figure 5D,G) and reduced sXBP1 expression (Figure 5A,C,I) and DNAJB9 expression (Figure 5E), whereas PIEZO1 expression remained unaltered (Figure 5F). We knocked down IRE1α using siRNA to determine whether the transcriptional suppression of IRE1α would similarly affect the PIEZO1‐mediated, FSS stimulation‐induced regulation of downstream pathways and MUC2 expression (Figure 5J,K). Consistent with the data obtained with the inhibitor, IRE1α silencing also inhibited the FSS stimulation‐induced upregulation of MUC2 expression and the associated changes in the levels of downstream molecules (Figure 5L–P). These results confirm that IRE1α is a key molecule that mediates PIEZO1‐regulated downstream sXBP1 activity and thereby influences MUC2 production.

**FIGURE 5:**
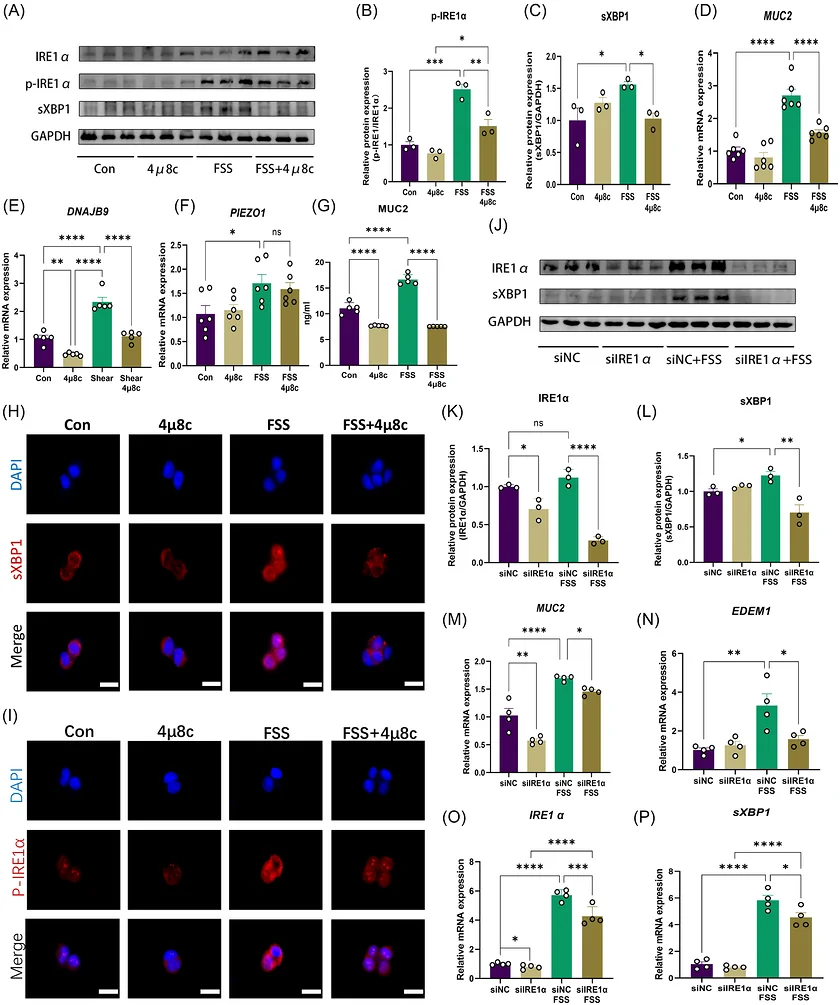
Effects of IRE1α inhibition or knockdown on the mechanically induced activation of the MUC2 production. (A–C) Representative Western blot images and corresponding quantitative analyses of phosphorylated IRE1α (p‐IRE1α/IRE1α) (A and B) and XBP1s (A and C) levels in cells cultured under FSS or no FSS conditions, with or without 4μ8c treatment (100 µmol/L, 24 h). (D–F) qRT–PCR analysis of MUC2 (D), DNAJB9 (E) and PIEZO1 (F) mRNA expression levels under the aforementioned experimental conditions. (G) ELISA results showing the MUC2 protein levels under the same experimental conditions as in (A–D). (H and I) Immunofluorescence staining for p‐IRE1α and sXBP1 (magnification: 400×; scale bar: 20 µm) in cells treated with or without FSS and 4μ8c. (J–L) Representative Western blot images and corresponding quantitative analyses of IRE1α (J and K) and sXBP1 (J and L) levels in cells cultured under FSS or no FSS conditions, with or without siIRE1α‐mediated IRE1α silencing. (M–P) qRT–PCR analysis of MUC2 (M), EDEM1 (N), IRE1α (O), and sXBP1 (P) mRNA expression levels in cells treated with or without FSS and siIRE1α‐mediated IRE1α knockdown. siIRE1α is small interfering RNA against IRE1α, transfected with Lipofectamine 3000 for transient gene knockdown. Means ± SEMs, *n* = 3–6. ns, not significant, *p < .05, **p < .01, ***p < .001, and ****p < .0001.

### 3.6 Result 6: PIEZO1 mediates Ca^2^^+^ influx to regulate endoplasmic reticulum calcium homeostasis, thereby modulating the IRE1α–XBP1 pathway and MUC2 production

Our above experiments confirmed that FSS stimulation regulated MUC2 production through the PIEZO1–IRE1α–XBP1 pathway. We explored whether PIEZO1 directly interacts with IRE1α by performing coimmunoprecipitation assays. The results revealed no direct physical interaction between IRE1α and PIEZO1 (Figure 6A), indicating that PIEZO1‐mediated changes in IRE1α activity likely occur via indirect mechanisms. As PIEZO1 functions as a mechanically gated cation channel, its activation profoundly affects cytoplasmic Ca^2^^+^ concentrations. Given that the endoplasmic reticulum is a primary Ca^2^^+^ storage organelle, PIEZO1 may regulate Ca^2^^+^ homeostasis in the ER by modulating cytoplasmic Ca^2^^+^ levels, thereby influencing the IRE1α–XBP1 pathway. For verification, ER calcium was labelled by transfection with the CMV‐ER‐LAR‐GECO1 plasmid, and cytosolic calcium was visualized with Fluo‐4 AM. Upon exposure to FSS, BAPTA‐AM was used to interrupt calcium trafficking between the cytosol and ER. Calcium levels, as well as alterations in the IRE1α pathway and MUC2, were further evaluated. Flow cytometry and Ca^2^^+^ indicator‐based fluorescence assays revealed that FSS stimulation increased both cytoplasmic and ER Ca^2^^+^ levels in LS174T cells. However, treatment with a Ca^2^^+^ chelator abrogated the FSS stimulation‐induced increase in cytoplasmic and ER Ca^2^^+^ concentrations (Figure 6D–G) and suppressed MUC2 production (Figure 6B,C). In addition, this intervention reversed the FSS stimulation‐induced upregulation of components of the IRE1α–XBP1 pathway (Figure 6H–J). To verify whether PIEZO1 directly modulates calcium levels under FSS stimulation, we performed calcium imaging assays following PIEZO1 inhibition with GsMTx4. As previously reported, FSS stimulation‐induced PIEZO1 activation increased cytosolic calcium levels, which was significantly reversed by GsMTx4.^5^ Importantly, we further observed that PIEZO1 inhibition altered the ER calcium content under FSS stimulation, indicating that PIEZO1 directly regulates both cytosolic and ER calcium homeostasis in response to mechanical stimulation (Figure S4a). Taken together, these findings indicate that FSS stimulation triggers PIEZO1‐mediated Ca^2^^+^ influx, which in turn regulates ER Ca^2^^+^ homeostasis and modulates the IRE1α–XBP1 pathway, ultimately governing MUC2 production. However, PIEZO1 is not the only mechanosensitive calcium channel expressed in goblet cells. Other mechanosensitive ion channels may also modulate calcium homeostasis and subsequently regulate the UPR. Accordingly, we reanalysed the RNA sequencing data and confirmed that PIEZO1 expression was significantly higher than that of other mechanosensitive ion channels in LS174T cells (Figure S4b). We then selected TRPA1 as a representative mechanosensitive calcium channel for functional assessment. Treatment with cinnamaldehyde, a specific agonist of TRPA1, did not notably effects on the UPR pathway or MUC2 production (Figure S4B–J). These findings are most likely attributed to the low expression of TRPA1 and other calcium‐permeable channels in LS174T cells, which conversely highlights the vital role of PIEZO1 in goblet cells.

**FIGURE 6:**
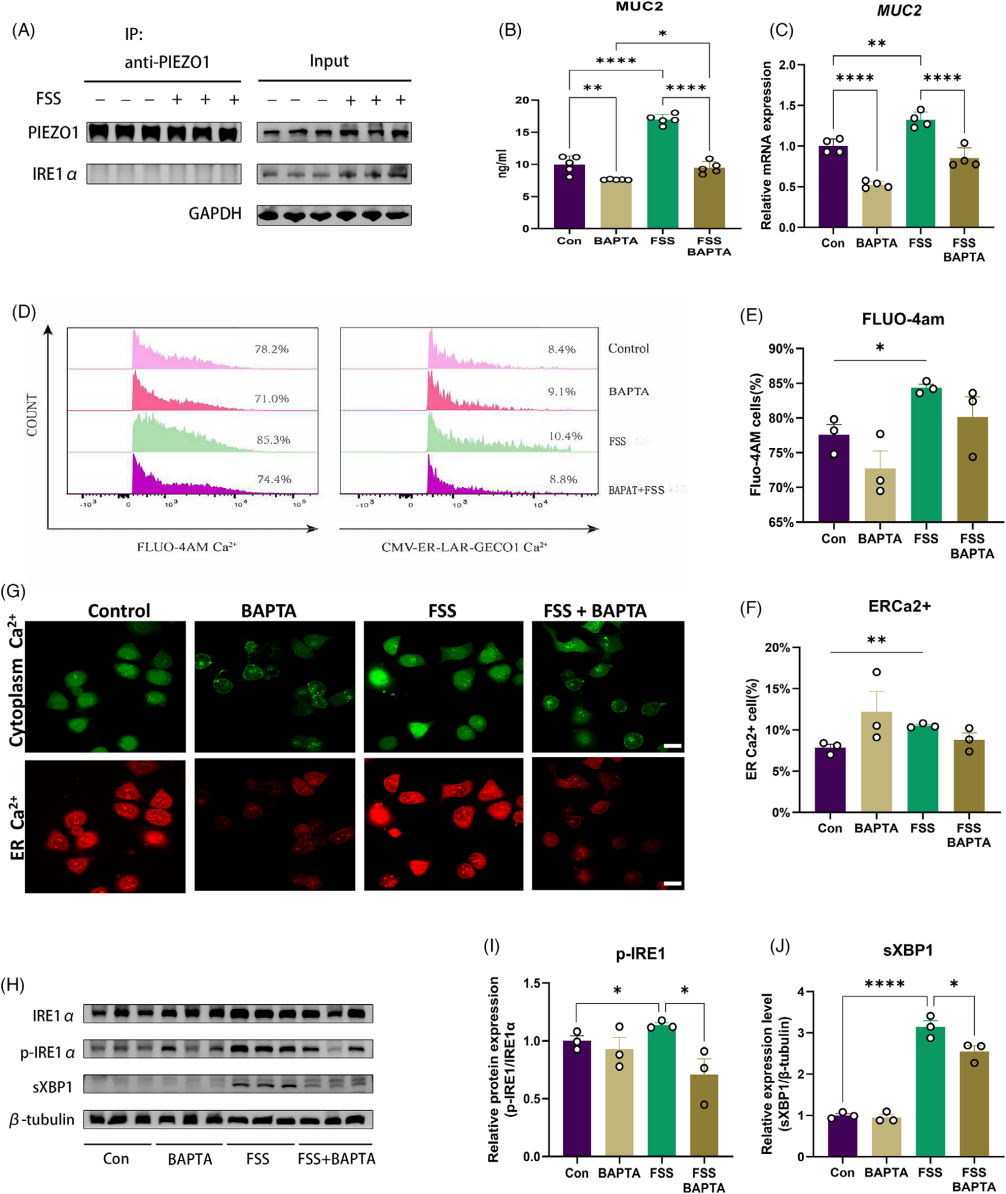
Effects of PIEZO1 activation on cytoplasmic and endoplasmic reticulum calcium levels. (A) CoIP assay showing the interaction between IRE1α and PIEZO1 in cells with or without FSS. (B and C) qRT–PCR analysis of MUC2 mRNA expression (B) and ELISA results for MUC2 protein levels (C) in cells cultured under FSS or no FSS conditions with or without BAPTA‐AM (10 µmol/L) treatment. (D–F) Flow cytometry analysis and corresponding quantitative results for cytoplasmic (D and E) and endoplasmic reticulum (ER) calcium (D and F) levels in cells treated with or without FSS and BAPTA‐AM. (G) Immunofluorescence staining showing cytoplasmic and ER calcium levels (magnification: 1000×; scale bar: 20 µm) in cells cultured under the aforementioned experimental conditions. (H–J) Representative Western blot images and corresponding quantitative analyses of phosphorylated IRE1α (p‐IRE1α/IRE1α) (H, I) and sXBP1 (H and J) levels in cells treated with or without FSS and BAPTA‐AM. The data from each group are shown as the means ± SEMs, *n* = 3–6. *p < .05, **p < .01, ***p < .001, and ****p < .0001.

### 3.7 Result 7. GC Piezo1 ablation aggravates DSS‐induced inflammation and exacerbates mucus barrier damage

DSS‐induced ulcerative colitis (UC) serves as a classic preclinical model to recapitulate severe intestinal mucus barrier disruption triggered by acute intestinal inflammation. To elucidate the biological function of goblet cell Piezo1 in colitis pathogenesis, we constructed three experimental groups: WT mice, DSS‐induced WT mice (WT‐DSS), and cKO‐DSS mice. Compared with WT‐DSS littermates, genetic deletion of Piezo1 specifically in goblet cells significantly exacerbated DSS‐induced pathological phenotypes. Specifically, GC Piezo1 ablation exacerbated progressive body weight loss, elevated disease activity index scores over the experimental time course, shortened colon length, and intensified histological inflammatory infiltration and injury in colonic tissues (Figure 7A–F). PAS staining further confirmed that compared with WT‐DSS control mice, cKO‐DSS mice exhibited much greater decreases in the thickness of the colonic mucus layer and pronounced mucus barrier breakdown (Figure 7G,H). In line with these observations, immunofluorescent staining for Muc2 demonstrated a striking reduction in the proportion of Muc2‐positive goblet cells within the colonic epithelium in the cKO‐DSS group (Figure 7I,J). SEM revealed severe morphological aberrations in colonic goblet cells upon Piezo1 deletion, characterized by cell swelling, exfoliation, and disordered ultrastructural architecture (Figure 7K). At the transcriptional level, RT–qPCR quantification revealed that Muc2 mRNA expression was further suppressed in cKO‐DSS colonic tissues (Figure 7L). Consistently, ELISA revealed markedly elevated secretion of the proinflammatory cytokines TNF‐α and IL‐1β in colonic homogenates from cKO‐DSS mice (Figure 8A,B). These results indicate that Piezo1 in goblet cells plays a crucial role in maintaining the stability of the intestinal mucus barrier in mice with DSS‐induced colitis.

**FIGURE 7:**
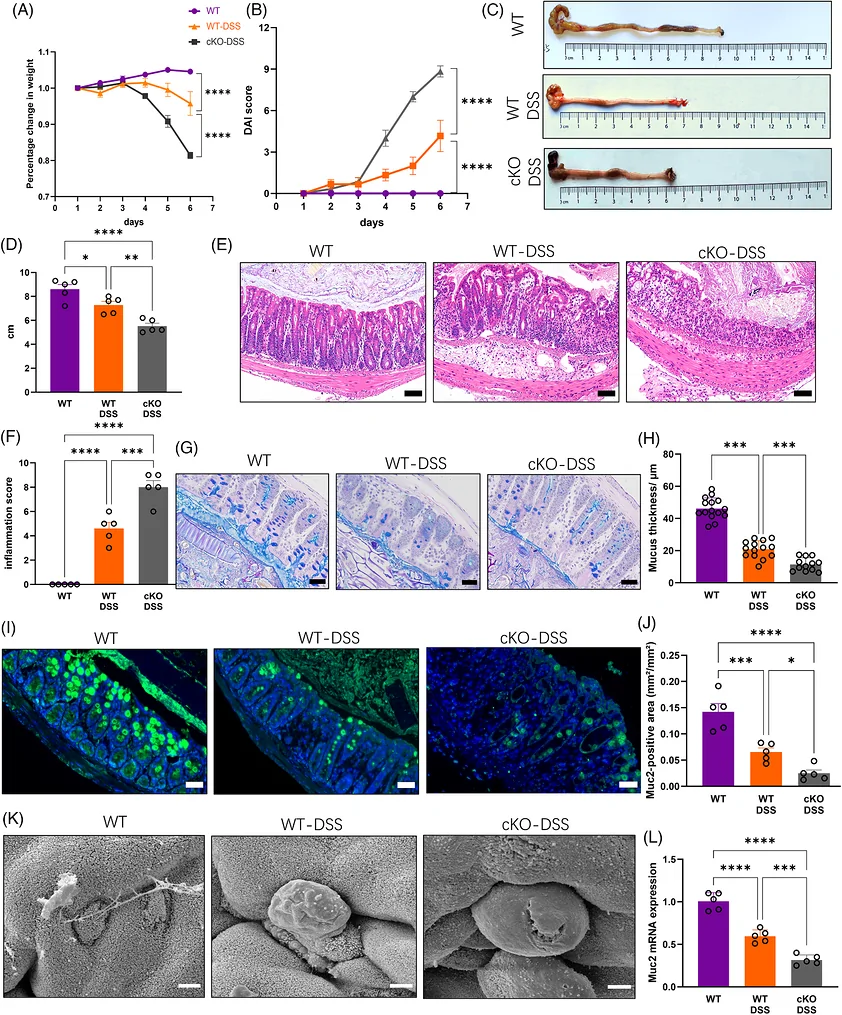
Pathological changes and mucus layer phenotypes in goblet cell‐specific Piezo1 knockout mice treated with DSS. (A) Dynamic changes in the body weight of WT, DSS‐treated WT and DSS‐treated cKO mice. (B) DAI scores of mice during the experimental period. (C and D) Representative images of the intestinal length (C) and corresponding quantitative analysis (D) from three groups of mice. (E and F) HE staining of colonic tissues (E, magnification: 100×; scale bar: 100 µm) and corresponding pathological scores and statistics (F) for WT, WT‐DSS, and cKO‐DSS mice. (G and H) AB–PAS staining of colonic tissues (G, magnification: 200×; scale bar: 50 µm) and quantitative analysis of the mucus layer thickness (H) from three groups of mice. Two to three representative images of the mucus layer were captured from each mouse for measurement (*n* = 5). (I and J) Immunofluorescence staining for Muc2 in colonic tissues (I, magnification: 200×; scale bar: 50 µm) and corresponding quantitative analysis (J) from three groups of mice (*n* = 5). (K) SEM images of colonic goblet cells from three groups of mice (magnification: 1000×; scale bar: 5 µm). (L) qRT–PCR analysis of Muc2 mRNA expression in the colonic goblet cells of WT, WT‐DSS, and cKO‐DSS mice. Means ± SEMs, *n* = 4–6. *p < .05, **p < .01, ***p < .001, and ****p < .0001.

**FIGURE 8:**
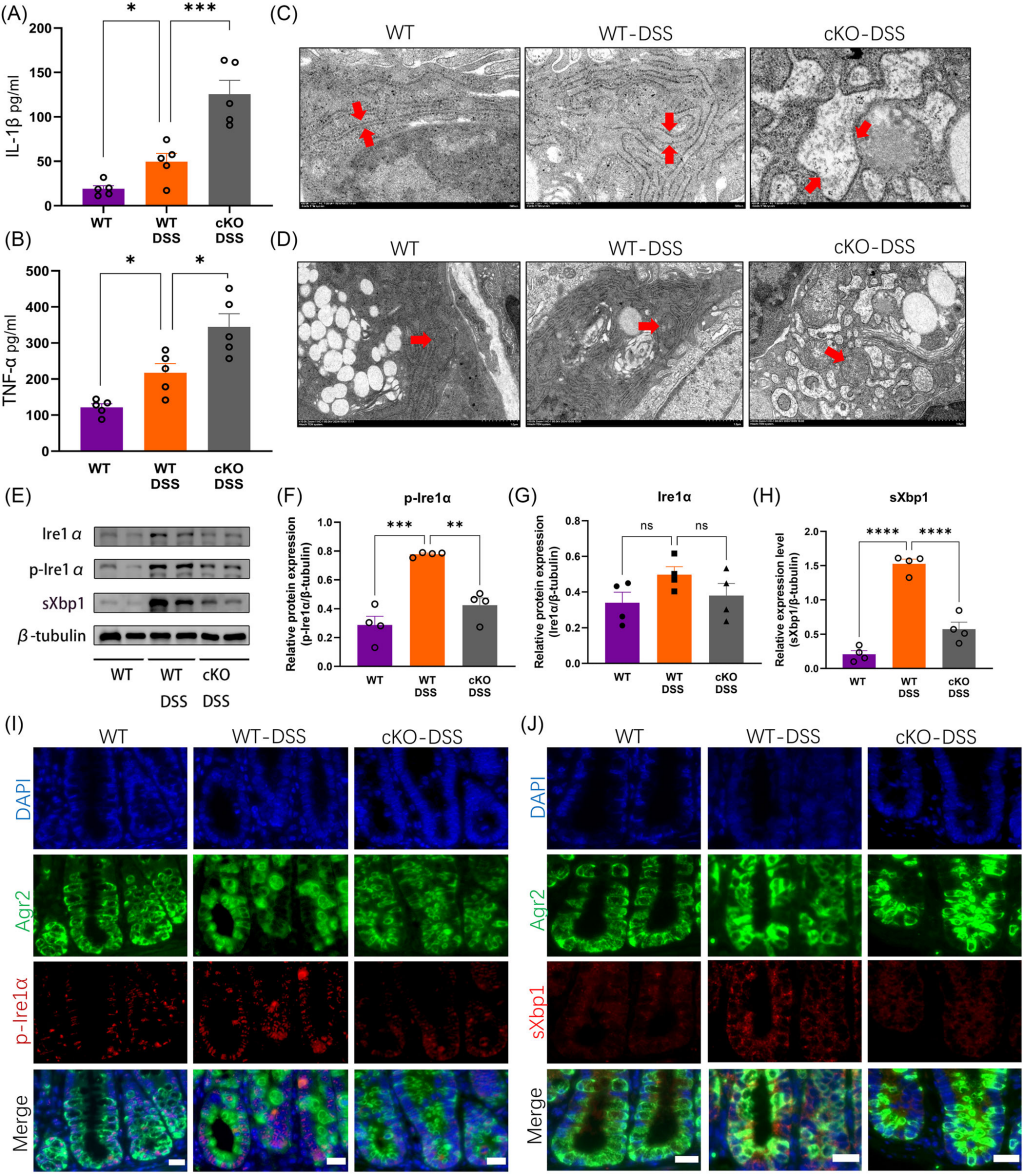
ER structure and the IRE1α–XBP1 pathway in DSS‐treated cKO mice. (A and B) ELISA results for IL‐1β (A) and TNF‐α levels (B) in the colonic tissues of WT, WT‐DSS, and cKO‐DSS mice. (C) Transmission electron microscopy images showing the endoplasmic reticulum structure of colonic goblet cells in WT, WT‐DSS, and cKO‐DSS mice (magnification: 30 000×; scale bar: 500 nm). (D) TEM images of mitochondrial structure in the colonic goblet cells of the three groups of mice (magnification: 10 000×; scale bar: 1 µm). (E–H) Representative Western blot images and corresponding quantitative analyses of phosphorylated Ire1α (p‐Ire1α) (E, F), total Ire1α (E and G), and sXbp1(E and H) levels in the colonic epithelial tissues of WT, WT‐DSS, and cKO‐DSS mice. (I and J) Images of immunofluorescence costaining for the goblet cell marker Agr2 with p‐Ire1α (I) and sXbp1 (J) in the colonic epithelial tissues of the three groups of mice (magnification: 200×; scale bar: 20 µm). Means ± SEMs, *n* = 4–6. ns, not significance, *p < .05, **p < .01, ***p < .001, and ****p < .0001.

### 3.8 Result 8. GC Piezo1 ablation aggravates DSS‐induced endoplasmic reticulum damage and suppresses the IRE1α–XBP1 pathway in mice

We subsequently explored functional changes in the endoplasmic reticulum by observing ER ultrastructure by transmission electron microscopy, and analysing the IRE1α signalling pathway by western blotting and immunofluorescence staining. Compared with DSS‐treated WT mice, DSS‐treated cKO mice exhibited more severe ER swelling (Figure 8C) and more pronounced mitochondrial crista loss (Figure 8D). To assess ER swelling, the area of the ER was quantified by electron micrographs, which revealed a significant increase in the ER area in cKO mice under the tested conditions (Figure S5a). Western blot analysis of scraped intestinal epithelial tissue samples revealed that DSS treatment upregulated phosphorylated Ire1α (p‐Ire1α) and sXbp1 protein levels, whereas these increases were attenuated in Piezo1‐knockout mice (Figure 8E–H). Immunofluorescence colocalization further confirmed that upon DSS stimulation, the knockout of Piezo1 in goblet cells significantly suppressed the stress‐induced activation of the IRE1α–XBP1 pathway (Figure 8I,J), thereby exacerbating intestinal mucus barrier impairment. Consistent with these findings, a recent study demonstrated that goblet cell‐specific sXbp1 knockout disrupts the mucus barrier, which in turn exacerbates intestinal inflammatory damage.^13^ These results reveal that Piezo1 knockout in goblet cells leads to dysregulation of the IRE1α–XBP1 pathway in the UPR, resulting in exacerbated endoplasmic reticulum damage.

### 3.9 Result 9. GC Piezo1 ablation aggravates mucus barrier and endoplasmic reticulum damage in a mouse model of irritable bowel syndrome

Given that the WAS‐induced IBS model recapitulates mild intestinal mucus barrier dysfunction under disorders of gut–brain interactions, we further explored whether similar changes occur in an IBS model. We established three experimental groups: WT mice, WT mice subjected to WAS, and cKO mice subjected to WAS. Compared with WT control mice, neither WAS‐exposed WT mice nor WAS‐treated cKO mice exhibited significant changes in body weight, intestinal length, or histological damage (Figure 9A–C,F), but both exhibited significantly greater sensitivity to the abdominal withdrawal reflex and pain threshold (Figure 9D,E), confirming the successful induction of the IBS model. Further analyses revealed that goblet cell‐specific Piezo1 knockout exacerbated WAS‐induced intestinal mucus barrier disruption (Figure 9G,H) and reduced Muc2 production (Figure 9I,J). Electron microscopy revealed that the goblet cells in the WAS‐treated cKO mice were swollen (Figure 9K) and presented obvious structural damage to the ER and an enlarged ER area (Figure 9L; Figure S5b). Subsequent Western blot analysis of scraped intestinal epithelial tissues revealed that phosphorylated Ire1α and sXbp1 were upregulated in WAS‐treated WT mice but downregulated in WAS‐exposed cKO mice (Figure 9M–Q), indicating impaired regulation of the UPR. Immunofluorescence colocalization assays further confirmed this trend for p‐Ire1α and sXbp1 levels in goblet cells (Figure 9R,S).

**FIGURE 9:**
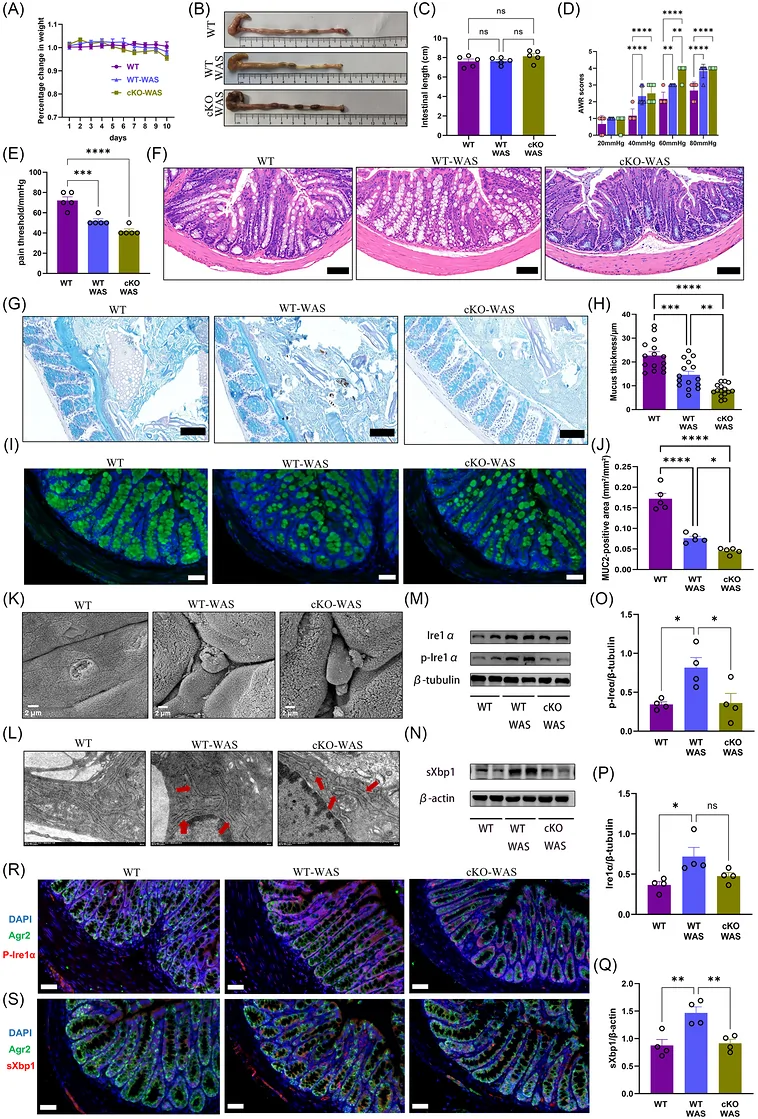
Mucus layer phenotypes, endoplasmic reticulum structure and the IRE1α–XBP1 pathway in goblet cell‐specific Piezo1 knockout mice treated with WAS. (A) Dynamic changes in the body weights of WT, WT‐WAS, and cKO‐WAS mice. (B and C) Representative images of the colon length (B) and corresponding quantitative analysis (C) in mice from three experimental groups. (D and E) AWR scores (D) and pain threshold scores (E) in mice from three experimental groups. (F) HE staining of colonic tissues (magnification: 200×; scale bar: 50 µm) from the mice. (G and H) AB–PAS staining of intestinal tissues (G, magnification: 200×; scale bar: 100 µm) and quantitative analysis of the mucus layer thickness (H) in mice from three experimental groups (*n* = 5). Two to three representative images of the mucus layer were captured from each mouse for measurement. (I and J) Immunofluorescence staining for intestinal Muc2 (I, magnification: 200×; scale bar: 50 µm) and corresponding quantitative analysis (J) in mice from three experimental groups(*n *= 5). (K) SEM images of colonic goblet cells from WT, WT‐WAS, and cKO‐WAS mice (magnification: 1000×; scale bar: 2 µm). (L) TEM images of the endoplasmic reticulum structure in the colonic goblet cells for three groups of mice (magnification: 20 000×; scale bar: 500 nm). (M–Q) Representative Western blot images and corresponding quantitative analyses of phosphorylated Ire1α(p‐Ire1α) (M, O), total Ire1α (M, P), and sXbp1 (N, Q) levels in colonic epithelial cells. (R and S) Images of immunofluorescence costaining for the goblet cell marker Agr2(green) with p‐Ire1α (R) and sXbp1 (S) in the colonic tissues of the mice (magnification: 200×; scale bar: 50 µm). Means ± SEMs, *n* = 4–6. ns, not significance, *p < .05, **p < .01, ***p < .001, and ****p < .0001.

Collectively, these results confirm that Piezo1‐mediated Ca^2^^+^ influx regulates MUC2 production by modulating ER Ca^2^^+^ homeostasis and the IRE1α–XBP1 pathway and that this process plays a conserved role in maintaining the intestinal mucus barrier in both IBS and ulcerative colitis models.

### 3.10 Result 10. Activation of Piezo1 improves the intestinal mucus barrier and alleviates structural damage in WAS/DSS mice

Our previous study demonstrated that goblet cell Piezo1 expression was decreased and that mucus barrier function was impaired in WAS mice. Intraperitoneal injection of Yoda1 alleviated behavioural abnormalities, restored intestinal mucus barrier integrity and reduced bacterial translocation in WAS mice, confirming the beneficial effects of Piezo1 activation in this model.^10^ On this basis, we administered Yoda1 intraperitoneally to DSS‐treated mice to explore the impacts of Piezo1 activation on the intestinal mucus barrier and colonic structural damage during acute DSS‐induced intestinal inflammation.

The results showed that Yoda1 treatment failed to reverse body weight loss in DSS mice, but effectively mitigated the alleviated DAI scores (Figure 10A,B). Moreover, Yoda1 administration reversed DSS‐induced colonic shortening and pathological damage (Figure 10C–F). AB‒PAS staining revealed that Yoda1 restored the reduced thickness of the colonic mucus layer in DSS‐treated mice (Figure 10G,H). Western blot analysis indicated that Piezo1 protein expression was downregulated in the colonic epithelium of DSS mice, and this reduction was markedly reversed by Yoda1 intervention (Figure 10I,J). Immunofluorescence colocalization assays further confirmed that Piezo1 expression in colonic goblet cells was suppressed under DSS stimulation, whereas Yoda1 treatment reversed this alteration (Figure 10K). Collectively, these findings suggest that Piezo1 activation ameliorates intestinal mucus barrier dysfunction and structural abnormalities in both WAS and DSS‐induced mouse models.

**FIGURE 10:**
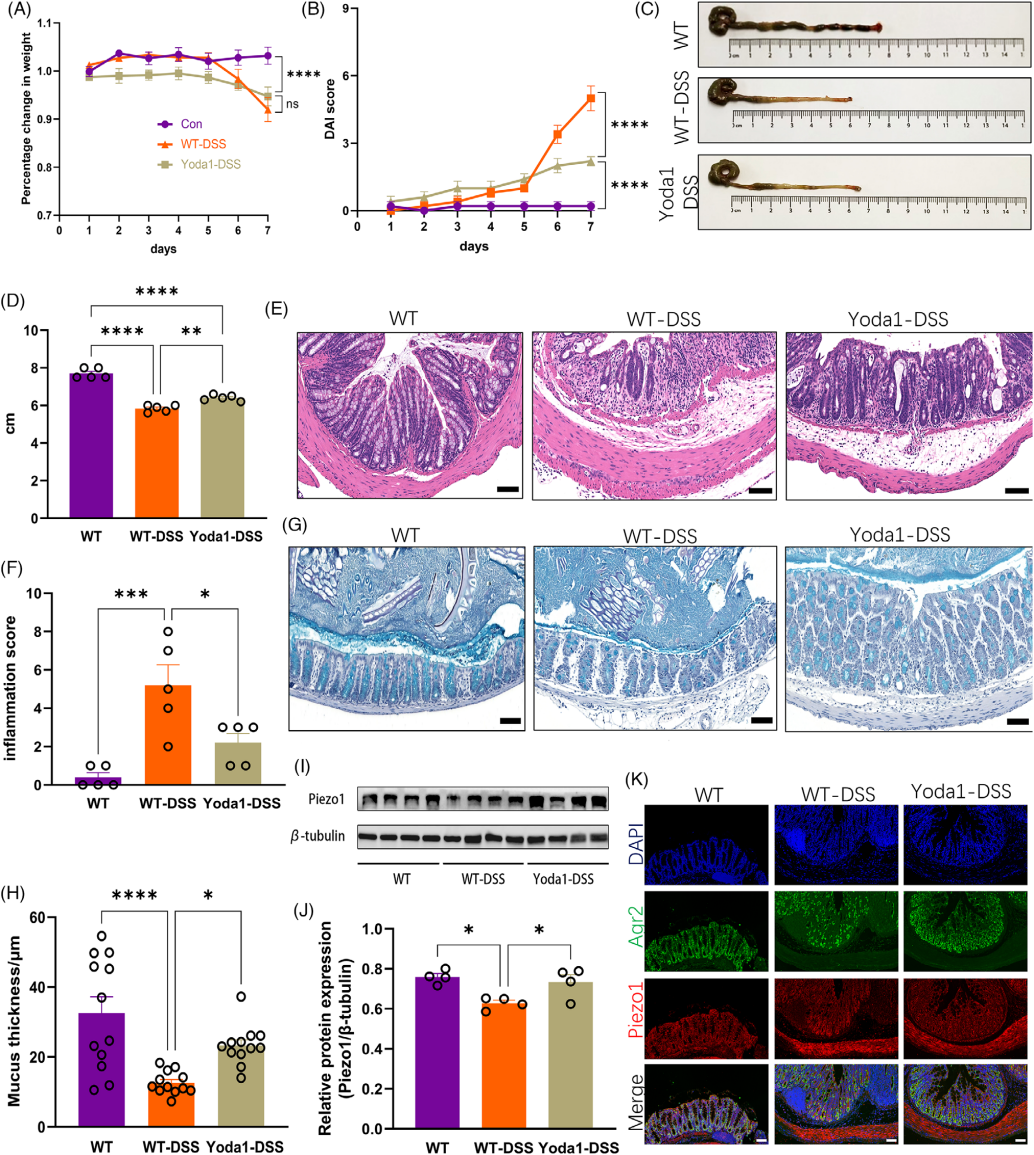
Mucus layer phenotypes and colonic pathological damage in DSS mice treated with Yoda1. (A) Dynamic changes in the body weights of WT, WT‐DSS, and Yoda1‐DSS. (B) DAI scores of mice from three experimental groups during the experimental period. (C and D) Representative images of the intestinal length (C) and corresponding quantitative analysis (D) of mice. (E and F) HE staining of colonic tissues (E, magnification: 200×; scale bar: 100 µm) and corresponding pathological scores and statistics (F) from the mice. (G and H) AB–PAS staining of intestinal tissues (G, magnification: 200×; scale bar: 100 µm) and quantitative analysis of the mucus layer thickness (H) in mice. Two to three representative images of the mucus layer were captured from each mouse for measurement. (I) Western blot detection of Piezo1 protein in colon tissues of WT, WT‐DSS and Yoda1‐DSS mice, with β‐tubulin as loading control; (J) Statistical quantification of relative Piezo1 protein levels. (K) Immunofluorescence co‐staining of Piezo1 (red), goblet cell marker Agr2 (green) and nuclear marker DAPI (blue) in colonic sections, as well as merged pictures (magnification: 200×; scale bar: 100 µm). Means ± SEMs, *n* = 4–5. ns, not significance, *p < .05, **p < .01, ***p < .001, and ****p < .0001.

## 4 DISCUSSION

Maintenance of intestinal mucus barrier integrity is widely recognized as a critical factor controlling the progression of IBD and IBS.^19^, ^20^ The results of this study reveal the mechanism regulating MUC2 production by goblet cells under physiological conditions: Piezo1 modulates calcium influx, which in turn regulates ER calcium homeostasis, controls the downstream IRE1α–XBP1 signalling pathway, and ultimately governs MUC2 production. In contrast, GC‐specific Piezo1 ablation disrupted ER homeostasis in mouse models of DSS‐induced colitis and WAS‐induced IBS, impaired MUC2 production, exacerbated mucosal barrier damage, and amplified inflammatory responses. Further experiments demonstrated that Piezo1 activation ameliorated intestinal mucus barrier defects and alleviated pathological lesions in DSS‐induced murine colitis models. Collectively, these data not only provide mechanistic insight into mucus barrier homeostasis under diverse gastrointestinal pathological states, but also identify Piezo1 as a critical upstream modulator of the IRE1α–XBP1‐dependent UPR that sustains intestinal barrier integrity. Importantly, our results highlight that Piezo1 agonism has promising translational therapeutic prospects for the clinical management of mucus barrier dysfunction‐related diseases, such as irritable bowel syndrome and ulcerative colitis.

Goblet cells secrete MUC2 through two functionally distinct modes: baseline constitutive secretion and stimulated secretion. Baseline constitutive secretion sustains mucus layer turnover, whereas stimulated secretion is triggered by mechanical forces, acetylcholine or bacterial products and can increase secretion by several thousand‐fold.^3^, ^21^ This secretion process is tightly coupled to ER function, as GCs rely on intact ER homeostasis to support the folding and trafficking of large, glycosylated MUC2 proteins. Goblet cells are enriched in genes that alleviate ERS, such as the autophagy regulator Beclin‐1, which enables the secretion of a thicker, less permeable mucus layer.^22^ However, the UPR, particularly its IRE1α–XBP1s branch, emerges as the dominant pathway for resolving ERS in GCs.

This study identifies a novel link between mechanical sensing via Piezo1 and UPR activation. Notably, the shaking‐induced fluid FSS stimulation adopted in our cellular experiments recapitulates a major physiological mechanical stimulus sensed by intestinal goblet cells in vivo. This experimental design connects our in vitro mechanistic observations to the pathophysiological context of intestinal disorders, supporting the biological relevance of Piezo1 as a key mechanotransducer that responds to gut luminal mechanical cues. Under physiological conditions, mechanical stimulation activates Piezo1, initiating calcium influx that increases ER calcium levels and triggering IRE1α–XBP1s upregulation. This cascade promotes MUC2 synthesis and production, which is consistent with prior reports that ER Ca^2^^+^ overload directly sensitizes the IRE1α–XBP1 axis to regulate cell fate and support survival.^23^ Piezo1‐mediated regulation of IRE1α may represent a selective unfolded protein response triggered by disrupted ER calcium homeostasis. ER Ca^2^^+^ overload redistributes endogenous GRP78 without altering its total protein abundance, weakens electrostatic interactions between GRP78 and the luminal domain of IRE1α, and facilitates the dissociation of the two proteins. Liberated IRE1α rapidly forms homodimers and undergoes autophosphorylation to initiate downstream signalling.^23^ In contrast, the GRP78‐binding domains of PERK and ATF6 are insensitive to ER Ca^2^^+^ fluctuations,^24^ leading to minimal activation of these two UPR branches. Upregulation of XBP1s, a critical downstream effector of IRE1α, modulates MUC2 production via two distinct mechanisms. First, as a potent transcription factor, XBP1s translocates to the nucleus to induce the synthesis of downstream MUC2 mRNA. Second, XBP1s can bind to endoplasmic reticulum stress response elements (ERSEs) and unfolded protein response elements (UPREs), thereby increasing the expression of genes involved in the ER machinery, improving the folding efficiency of MUC2 within the ER, and ultimately promoting MUC2 protein expression.^25^ In the present study, the upregulation of sXBP1 was accompanied by increased MUC2 mRNA and protein levels, suggesting that both mechanisms may contribute to MUC2 regulation. Previous studies have demonstrated that XBP1 inhibits IRE1β‐mediated Muc2 mRNA decay through AGR2 induction, thereby positioning XBP1 upstream of MUC2 secretion.^13^, ^26^, ^27^ Our results provide important insights into how cells sense mechanical forces to regulate XBP1, thereby influencing the downstream production of MUC2.

In both murine models of IBD and IBS, GCs resulted in significant ER damage. This damage correlated with impaired mucus barrier function, and this observation is consistent with the extensive literature that connects ER stress to mucus dysfunction in individuals with IBD.^28^, ^29^, ^30^, ^31^ Our data revealed that Piezo1 expression is downregulated while the IRE1α–XBP1 axis is concomitantly upregulated in both DSS‐induced colitis and WAS‐induced stress models. The upregulation of IRE1α–XBP1 is likely driven by alternative stress stimuli to modulate MUC2 production. Activated IRE1α–XBP1 signalling enhances ER protein‐folding capacity, eliminates misfolded mucin proteins, and facilitates the proper processing and trafficking of mature MUC2, thereby counteracting excessive ER stress. Further genetic evidence has demonstrated that goblet cell‐specific Piezo1 ablation impairs the activation of the IRE1α–XBP1 pathway. Defective signalling of this branch abolishes the adaptive protective effects of the UPR, which consequently exacerbates intestinal barrier disruption and increases the production of proinflammatory cytokines. Conversely, pharmacological activation of Piezo1 restored intestinal mucus barrier homeostasis and ameliorated barrier defects in the context of DSS and WAS. These findings align with prior work showing that disruption of stress‐induced IRE1α/XBP1 upregulation in cortical neurons increases apoptosis,^32^ highlighting the conserved protective role of the IRE1α–XBP1 pathway across cell types. Moreover, we also found that Piezo1 knockdown exacerbates the loss of mitochondrial cristae in DSS‐treated mice. This phenomenon may be a downstream effect of ER damage, or alternatively, Piezo1 depletion may directly impair the basic functions of mitochondria. Collectively, these data establish that Piezo1 in GCs is indispensable for maintaining ER function, sustaining UPR‐mediated ERS resolution, and preserving MUC2 production, which are the core processes that prevent intestinal inflammation.

The shared hallmark of mucus barrier dysfunction in IBD and IBS underpins the clinical relevance of our work. Diminished mucus integrity permits luminal microbiota translocation, triggers excessive inflammatory signalling, and amplifies intestinal mucosal injury—pathological processes that are well documented in both disorders.^33^ Consistent with these findings, mice with inherent mucus layer defects exhibit increased susceptibility to DSS‐induced colitis, highlighting mucus deficiency as a key driver of ulcerative colitis pathogenesis.^34^ Our previous work has revealed that Piezo1 activation reinforces mucus barrier function and restricts bacterial infiltration in WAS model mice.^10^ The results of the present study further demonstrated that Piezo1 stimulation likewise improved mucus barrier integrity and alleviated pathological damage to the colon in DSS‐induced colitis mice. Collectively, these data indicate that Piezo1 activation exerts a pivotal protective effect against both mild mucus barrier impairment triggered by WAS and acute barrier damage elicited by DSS, indicating that Piezo1 is a promising target with great translational potential for future clinical application.

While our genetic and functional studies revealed a critical role for the Piezo1–ER calcium–IRE1α–XBP1–MUC2 axis in mucus barrier regulation, several limitations warrant consideration. First, the canonical goblet cell line LS174T was used as the primary cellular model in this study, which cannot fully recapitulate the biological characteristics of primary goblet cells. Intestinal organoids may serve as a superior experimental platform for subsequent investigations. One additional consideration is that definitive validation of the ER localization of the sensor via costaining with a canonical ER marker remains an important avenue for future investigation. We plan to validate this colocalization in our ongoing and future studies, which we believe will further strengthen the conclusions of the present work. Second, although prior studies have confirmed a link between increased ER calcium levels and IRE1α–XBP1s activation,^23^ recent research has indicated that calcium‐mediated calreticulin‐IRE1α interactions trigger dynamic fluctuations in IRE1α activity.^35^ However, the exact molecular cascade bridging ER calcium flux and IRE1α activation has not yet been fully elucidated and requires additional exploration. Third, the upregulation of the IRE1α–XBP1 pathway promoted MUC2 mRNA synthesis and increased MUC2 protein levels in the present study. However, the alterations in mRNA synthesis and protein folding capacity mediated by this pathway warrant further investigation. Existing evidence has demonstrated that the endoribonuclease activity of IRE1β is significantly lower than that of IRE1α and that IRE1β plays an indecisive role in XBP1 production.^23^, ^27^ Notably, the endoribonuclease activity of IRE1β can degrade MUC2 mRNA and contribute to the homeostasis of intestinal mucins.^13^ The role of IRE1β in the Piezo1‐mediated pathway was not addressed in the current research. Whether IRE1β merely regulates Muc2 mRNA decay downstream of XBP1 or integrated into Piezo1‐dependent UPR activation upon mechanical stimulation remains unresolved. Future studies addressing these research gaps are crucial for comprehensively mapping the transcriptional and signalling networks controlled by Piezo1 and IRE1β in GCs. Notably, we comprehensively characterized the functional Piezo1‐Ca^2^^+^‐IRE1α cascade that governs mucus production in goblet cells under physiological conditions. Nevertheless, our mechanistic evidence is derived from in vitro cellular experiments, which may not fully recapitulate the complex pathological microenvironment in diseased animal models. Nevertheless, genetic ablation and pharmacological activation of Piezo1 in animal models produced opposing phenotypes of aggravated and alleviated intestinal mucus barrier damage, strongly demonstrating the essential contribution of Piezo1‐governed signalling pathways to intestinal disease pathogenesis. Future investigations are needed to develop optimized strategies for targeted activation of goblet cell Piezo1 in disease contexts to restore mucus barrier integrity, thereby facilitating subsequent clinical translation.

In conclusion, our findings reveal that goblet cell Piezo1 maintains intestinal mucus barrier homeostasis by regulating MUC2 production via the ER calcium–IRE1α–XBP1s pathway. These results were further validated in murine models of IBD and IBS. This work not only advances our understanding of the role of mechanical sensing in regulating the gastrointestinal barrier but also identifies Piezo1 and XBP1 as promising therapeutic targets for IBD, IBS, and other diseases driven by mucus barrier impairment.

## AUTHOR CONTRIBUTIONS

Jiandi Wu: conceptualization, data curation, investigation, methodology, validation, formal analysis, writing‐original draft. Shaotong Wang: data curation, investigation, validation, formal analysis, writing‐original draft. Zhe Wang, Jianhua Fang, Xueyan Li, QingQing Zhang, Yan Dai, Wenjing Tian and Yize Ma: investigation, validation, formal analysis. Yudong Jiang, Lei Zhang, Gangping Li, Xiaohua Hou and Jun Song: formal analysis, resources, supervision, writing‐review & editing, project administration, funding acquisition. All authors have reviewed and endorsed the final draft of the manuscript.

## ETHICS STATEMENT

All experimental procedures were approved by the Experimental Animal Ethics Committee of Huazhong University of Science and Technology (Approval No. [2024] IACUC number: 4909).

## CONFLICT OF INTEREST STATEMENT

The authors declare no conflicts of interest.

## Supporting information



Supporting Information

## Data Availability

The data are available from the corresponding author on reasonable request.
