# Supplementary material for: Piezo1‐mediated unfolded protein response in goblet cells regulates intestinal mucus barrier homeostasis through the Ca2 +–IRE1α–XBP1 pathway
Source: Clin Transl Med. 2026 Oct 27;16(11):e70886. doi: 10.1002/ctm2.70886 (PMC13714353; doi:10.1002/ctm2.70886)
Supplement: Supplementary file 1 [file CTM2-16-e70886-s001.docx]

**Supplementary Information:**

DAPI

WT

cKO

ZO-1

Merge

**Supplementary Fig. 1: Alterations in colonic epithelial barrier function in GC-specific Piezo1 knockout mice (cKO).**

Immunofluorescence staining for ZO-1 in colon tissues; Scale bar: 50 µm, 200× magnification.

**c**

**a**

**b**

**d**

**e**

**Supplementary Fig. 2: Alterations in the PERK and ATF6 signaling pathways under FSS or Yoda1 treatment.**

(a) Relative mRNA expression level of GRP78 measured by qRT–PCR. (b) Relative mRNA expression level of PERK measured by qRT–PCR. (c) Relative mRNA expression level of eIF2α measured by qRT–PCR. (d) Relative mRNA expression level of CHOP measured by qRT–PCR. (e) Relative mRNA expression level of ATF6 measured by qRT–PCR. ns, not significant, *p < 0.05, **p < 0.01, ***p < 0.001, ****p < 0.0001.

**b**

**a**

**d**

**c**

**Supplementary Fig. 3: Specific Western blot bands for GRP78, XBP1 and CHOP detected in tunicamycin-treated LS174T cells**

(a) Western blot images showing GRP78, XBP1s and CHOP expression in LS174T cells treated with tunicamycin (2 µg/mL, 24 h). (b–d) Relative protein quantification of GRP78, XBP1s and CHOP; *p < 0.05, **p < 0.01.

FSS+GsMTx4

Control

GsMTx4

FSS

Cytoplasm Ca^2+^

ER Ca^2+^

**a**

**b**

**c**

**d**

**e**

**f**

**g**

**h**

**i**

**j**

**Supplementary Fig. 4: Expression and impacts of Piezo1 and other calcium channels in LS174T cells.**

(a) Immunofluorescence staining showing cytoplasmic and ER calcium levels (magnification: 1000×; scale bar: 20 μm) in cells subjected to shear stress with or without GsMTx4 intervention; (b) Relative gene expression (FPKM values) of Piezo1 and multiple calcium-permeable ion channels/receptors (TRPV1, TRPV3, TRPV6, TLR4, TRPA1, TRPC1, TRPC6) in LS174T cells; (c–j) Relative mRNA expression levels of Mucin2, IRE1α, XBP1s, GRP78, ATF6, PERK, eIF2α and CHOP in LS174T cells under control (Con) and cinnamaldehyde (TRPA1 agonist) treatment groups. ns, not significant, ****p < 0.0001.

**b**

**a**

**Supplementary Fig. 5: The area of the endoplasmic reticulum in animal models.**

(a) TEM-based scatter analysis of ER area versus cytoplasmic area in colonic goblet cells from WT, WT-DSS and cKO-DSS mice; dot size corresponds to the ER-to-cytoplasm area ratio. (b) TEM-based scatter analysis of ER area versus cytoplasmic area in colonic goblet cells from WT, WT-WAS and cKO-WAS mice; dot size corresponds to the ER-to-cytoplasm area ratio.
